# Evaluating the properties of the fragility index of meta-analyses

**DOI:** 10.1186/s12874-025-02648-5

**Published:** 2025-09-25

**Authors:** Aiwen Xing, Xing Xing, Mohammad Hassan Murad, Lifeng Lin

**Affiliations:** 1 Biostatistics and Research Decision Sciences, Merck & Co., Inc, Rahway, NJ USA; 2 Department of Biostatistics, Johns Hopkins Bloomberg School of Public Health, Baltimore, MD USA; 3 Evidence-Based Practice Center, Mayo Clinic, Rochester, MN USA; 4 Department of Epidemiology and Biostatistics, University of Arizona, 1295 N. Martin Ave., Tucson, AZ 85724 USA

**Keywords:** Evidence synthesis, Fragility index, Heterogeneity, Meta-analysis

## Abstract

**Background:**

The fragility index (FI) has become an increasingly popular supplementary measure for evaluating the robustness of a study’s conclusions. While initially developed for individual clinical trials, the FI has been extended to meta-analyses (MAs) of multiple studies. However, the existing literature provides limited insights into the properties of the FI in the context of MAs. This study aims to explore various statistical methods for MAs and assess the improvement in FI of MAs compared to the individual studies they comprise.

**Methods:**

We investigated the empirical distributions of FI and fragility quotient (FQ) using a large database of Cochrane MAs with binary outcomes. The FI of MAs was calculated under different statistical methods, including fixed-effect and random-effects models, with between-study variance estimators (restricted maximum-likelihood and DerSimonian–Laird), alongside Hartung–Knapp–Sidik–Jonkman (HKSJ) confidence interval adjustments. Subgroup analyses were performed to explore the impact of heterogeneity, sample size, and effect measures on fragility. Furthermore, we employed a metric to evaluate the improvement in fragility by comparing the FI of MAs with the FIs of the individual studies they included.

**Results:**

The median FI was 5 (IQR: 2–11) among 3,758 MAs analyzed, with 29% reporting statistically significant results. Notably, 15% of MAs had an FI of 1, and 54% had an FI ≤ 5. MAs with larger sample sizes or higher ${I}^{2}$ values, tended to exhibit greater robustness. HKSJ adjustments introduced more uncertainty, yielding more fragile results compared to analyses without these adjustments. Fragility improvement was higher in MAs with considerable heterogeneity.

**Conclusions:**

This study highlights the variability in fragility across MAs and underscores the influence of heterogeneity and statistical methods on FI. Further research is warranted to refine the assessment of fragility and incorporate clinical relevance into these evaluations.

**Supplementary Information:**

The online version contains supplementary material available at 10.1186/s12874-025-02648-5.

## Background

Meta-analysis (MA) is a widely used method for synthesizing evidence from multiple individual studies to support decision-making in healthcare [1–4]. By combining findings across studies, MAs typically provide effect estimates with greater precision than those derived from individual studies alone [5, 6]. A critical component of MA is the investigation and assessment of treatment effect heterogeneity [7–9], which reflects differences in the true underlying effects among the included studies [10, 11]. Heterogeneity can greatly influence the reliability and generalizability of the synthesized results [12–14].

Recently, many concerns have been raised in the literature about research reproducibility and replicability. A novel metric called the fragility index (FI) [15] was proposed to quantitatively measure the robustness of the results of randomized controlled trials (RCTs) with binary outcomes in terms of statistical significance. The FI is defined as the minimum number of event status modifications of patients needed to change the significance of the results. Later, this concept was extended to MA [16], which was defined as the minimum number of changes in the event status of patients across the included studies that would alter the significance of the synthesized treatment effect. In addition, due to concerns about the correlation between FI and trial size [17–20], a relative metric called fragility quotient (FQ) [21, 22], the absolute value of FI divided by the total sample size, was also proposed to address the issue of trial size.

In deriving the FI of an MA, statistical significance is determined by whether the synthesized treatment effect’s 95% confidence interval (CI) includes the null effect [20]. Consequently, the choice of statistical methods for calculating synthesized effects and their corresponding 95% CIs plays a critical role in determining fragility. Furthermore, variability in the true underlying effects across included studies is a common feature of MAs [23, 24]. Recognizing this heterogeneity informs clinical decision-making for researchers and clinicians [11, 23], emphasizing the importance of investigating uncertainty to assess the reliability and robustness of the integrated results. The methods used to estimate heterogeneity can significantly influence the overall effect estimates and their CIs [25]. To the best of our knowledge, there is very limited research examining how heterogeneity, including its evaluation, impacts the FI of MAs.

This study investigates the empirical distributions of FI using a large database of MAs from the Cochrane Library. It summarizes and compares several commonly used statistical methods in MA that may influence the FI. Furthermore, it introduces a metric to evaluate fragility improvement by comparing the FI of MAs with the FIs of the individual studies included in the MAs.

## Methods

The reporting of this study follows the guidance about reporting meta-epidemiological research [26].

### FI and FQ of MAs

Recent literature has criticized the misinterpretation and misuse of *P*-values by researchers when interpreting study results [27–32]. To aid in the interpretation of clinical findings, the FI was introduced as a novel metric to supplement *P*-values in assessing the robustness of treatment effects in clinical studies with binary outcomes [15]. The FI is defined as the minimum number of event status modifications required to alter the statistical significance of an RCT.

The concept of FI has since been extended to pairwise MAs [16], where it is similarly defined as the smallest number of event status changes across all included trials needed to alter the statistical significance of the pooled effect. In the case of MAs, the FI is computed using an iterative search algorithm that explores different combinations of event flips across treatment and control arms, always in the direction that moves the pooled estimate toward the null. For significant MAs, modifications occur in one arm to shift the synthesized treatment effect towards the null value, ensuring an optimal solution for calculating the FI.

The FI is an “absolute” value that does not account for sample size. To address this limitation, a complementary measure, FQ, defined as the FI divided by the total sample size, has been proposed to enable comparisons across studies. For MAs, the FQ is similarly defined as the FI divided by the total sample size of all included studies. The FQ of an MA can be interpreted as the proportion of event status modifications required across all studies in the MA to alter the statistical significance of the synthesized relative effect.

### Statistical methods for MAs

#### MA models

Consider an MA including $N$ studies with binary outcomes; each study compares two groups, denoted by 0 and 1, where 0 denotes the control and 1 the treatment groups. The event counts and the total sample sizes are reported as ${e}_{i0}$, ${e}_{i1}$, ${n}_{i0}$, and ${n}_{i1}$ for study $i$ ($i=1, 2, \dots , N)$. Various effect measures can be used to quantify treatment effects with binary outcomes, including odds ratio (OR), risk difference (RD), and relative risk (RR). The OR and RR can be analyzed based on the logarithmic scale. The continuity correction of 0.5 is usually applied to all studies with zero counts [33].

Two approaches are commonly used to synthesize study findings from an MA: the fixed-effect (FE) model (also known as the common-effect model) and the random-effects (RE) model. Let ${y}_{i}$ be the estimated effect size and ${s}_{i}$ be the standard error in study $i$. Assuming the estimated effect size following the normal distribution ${y}_{i}\sim N\left({\theta }_{i},{s}_{i}^{2}\right)$, where ${\theta }_{i}$ denotes the underlying study-specific true effect size of study $i$. The within-study standard errors ${s}_{i}$ are assumed to be a known value.

In the FE model, all studies in the MA share one common underlying true effect size, i.e., ${\theta }_{i}=\theta$. All variability in the estimated effects is attributed solely to sampling error. However, studies often differ in design, participants, location, and methods of measuring treatment effects [14, 34]. This diversity inevitably leads to heterogeneity, making the use of the FE model less plausible [11, 35, 36].

To account for heterogeneity, the RE model is more commonly employed in the MA literature [34, 35]. In the RE model, the underlying true effect sizes are assumed to vary across studies. The variability in the true effect sizes arises from both within-trial variance (i.e., sampling error) and between-trial variance. Effect sizes in the studies are treated as a random sample from a distribution, and the goal is to estimate the mean of this distribution [14]. The underlying true effect sizes are typically assumed to follow a normal distribution, ${\theta }_{i}\sim N(\theta ,{\tau }^{2})$, where ${\tau }^{2}$ represents the between-study variance due to heterogeneity.

#### Between-study heterogeneity

The assessment of between-study heterogeneity is crucial in MAs [23, 24]. A reliable estimate of the heterogeneity variance ${\tau }^{2}$ provides a solid foundation for the synthesized results in MAs. Many methods are available for estimating ${\tau }^{2}$ [14, 35]. Among them, the method of moments estimator, DerSimonian–Laird (DL) [37], is perhaps the most widely used. The DL method is a non-iterative algorithm that is straightforward to implement [14]. Due to these properties, it is the default method in some popular meta-analytic software packages, such as RevMan [38].

However, the DL method has been criticized for introducing significant bias and producing narrow CIs for effect estimates [38–41]. To address these limitations, a variety of alternative methods have been developed [42], including restricted maximum-likelihood (REML) [43] and estimators proposed by Paule and Mande [44], Sidik and Jonkman [45, 46], and Hartung and Makambi [47]. REML, in particular, is recommended for its higher precision and will be primarily considered in this study [48]. It uses an iterative algorithm [43], with estimates derived by maximizing the log-restricted likelihood function [49]. Nevertheless, the algorithm for REML may fail to converge, resulting in errors and missing estimates.

Different methods for estimating ${\tau }^{2}$ can yield notably different results [12, 25, 45, 48], including varying degrees of uncertainty and even disagreements regarding statistical significance. Therefore, the choice of heterogeneity variance estimators is a crucial consideration in MAs.

#### ${I}^{2}$ statistic and $Q$ test

In the current literature, several well-known methods are available to assess the presence of between-study heterogeneity. The chi-squared homogeneity test [50], also known as the $Q$ test [51], is a classical statistic frequently reported in most published MAs. It evaluates whether the observed differences in study results are due solely to chance [13].

However, the $Q$ test has notable limitations. First, while it identifies the presence of heterogeneity, it does not quantify its extent [13]. Additionally, the $Q$ test has low power when only a few studies are included, yet excessive power to detect trivial heterogeneity when many studies are involved [7, 52]. Moreover, the $Q$ test depends on the sample size and the magnitudes of effect sizes, making it unsuitable for comparing heterogeneity levels across MAs.

To address these issues, the ${I}^{2}$ statistic [10] was introduced to quantify the proportion of variation attributable to heterogeneity (between-study variance ${\tau }^{2}$) as opposed to sampling error (within-study variance). It has become a widely used metric in the MA literature for assessing heterogeneity. An empirical guideline [53] has been proposed to roughly interpret the magnitudes of ${I}^{2}$: $0\le {I}^{2}\le 0.4$ indicates that heterogeneity might not be important; $0.3\le {I}^{2}\le 0.6$ may represent moderate heterogeneity; $0.5\le {I}^{2}\le 0.9$ may represent substantial heterogeneity; and $0.75\le {I}^{2}\le 1$ implies considerable heterogeneity [53]. However, various factors can influence the interpretation of ${I}^{2}$, including the scale and direction of effect sizes and the strength of evidence for heterogeneity. Given these factors, overlapping ranges of ${I}^{2}$ are likely more reasonable, as strict thresholds may lead to misleading conclusions.

#### CI adjustments

The traditional CI in an MA is based on a normal distribution, while various approaches have been proposed to improve the CI estimation. In particular, the Hartung–Knapp–Sidik–Jonkman (HKSJ) method [45, 54] is highly recommended. It is based on a $t$-distribution and uses an alternative weighted variance for the summary estimates. Several simulation studies have demonstrated that the HKSJ method effectively yields more appropriate CI coverage probabilities [48, 54], especially when the number of included studies is small.

### Improvement in the FI of an MA

One of our aims is to evaluate the improvement in fragility by comparing the FI of MAs with the FIs of the individual studies they include. To achieve this, we used a metric defined as the proportion of cases where the FI of the MA is more robust than the FIs of the individual studies. Specifically, the improvement is calculated as the total number of individual studies with an FI smaller (and thus more fragile) than that of the MA, divided by the total number of studies. The improvement proportion is expressed as ${N}^{-1}\sum_{i=1}^{N}I\left({\text{FI}}_{i}<{\text{FI}}_{\text{MA}}\right)\times 100\%$, where $N$ is the number of individual studies. Here, $I\left(\cdot \right)$ is an indicator function equalling 1 when the FI of a specific study $i$ is smaller than that of the MA and zero otherwise.

The improvement proportion is higher when the FI of the MA exceeds the FIs of more individual studies, indicating that the MA result is more robust. We classified the improvement proportion into four levels: no improvement (0%), slight improvement (between 0 and 50%), considerable improvement (between 50 and 100%), and complete improvement (100%).

### Data sources

We collected MAs with binary outcomes from the Cochrane Library, a comprehensive resource providing valuable healthcare-related information to support decision-making. All Cochrane reviews published from 2003 (Issue 1) to 2020 (Issue 1) were included. If a review had multiple updates during this period, only the latest version was retained, and withdrawn reviews were excluded. Further details on the data collection process are available in our previous publications [55–57].

Since MAs from the same systematic reviews likely include data from overlapping studies and populations, we selected only the largest MA within each systematic review to avoid redundancy. Additionally, restricting the analysis to the largest MA reduces computation time for the iterative algorithm used to derive the FI, compared to analyzing all MAs in the database exhaustively.

### Data analyses

We evaluated the FI and FQ for MAs using several commonly applied methods: the FE model and the RE model based on two between-study variance estimators, REML (selected for its superior performance) and DL (chosen for its widespread use), along with the HKSJ CI adjustment. Specifically, we applied the FE model and four scenarios of the RE model for FI computation: 1) REML with HKSJ; 2) REML without HKSJ; 3) DL with HKSJ; and 4) DL without HKSJ. These scenarios are referred to as scenarios 1 through 4 in the following.

For the main analysis, we focused on the empirical distribution of FI based on the scenario of REML with HKSJ (scenario 1). Results for the FE model and the other three scenarios are presented in the Supplementary Materials. Given its advantages in estimating ${\tau }^{2}$ with higher precision and better statistical properties, REML was selected as the primary heterogeneity estimator in this study. Despite its potential convergence issues in small or sparse MAs, REML remains a widely recommended approach for robust meta-analytic inference, and therefore, forms the foundation for our main analysis scenario. Additionally, we focused on MAs with statistically significant results, as this aligns with the original motivation behind the development of FI, reflecting the common emphasis of researchers on achieving statistical significance. All analyses were conducted using a statistical significance level of 0.05. The analyses were implemented with the R package “fragility” (version 1.0) in R (version 3.5.3) [58].

We also explored the impacts of total sample size, total number of events, ${I}^{2}$, effect measure, and effect magnitude on the FI of MAs. Total sample sizes were grouped into six levels: < 50, 50–100, 100–200, 200–500, 500–1000, and ≥ 1000. The total numbers of events were similarly categorized into six levels: < 10, 10–50, 50–100, 100–500, 500–1000, and ≥ 1000. The cutoffs for ${I}^{2}$ were based on the Cochrane Handbook [53]. Overlapping ranges were included to account for uncertainties in interpreting ${I}^{2}$.

We considered three effect measures: OR, RR, and RD. For effect magnitude, to standardize comparisons, we converted OR to 1/OR when OR < 1. The magnitudes of OR were categorized according to a method [59] consistent with Cohen’s $d$ [60]: 1–1.68 (small), 1.68–3.47 (moderate), 3.47–6.71 (median), and > 6.71 (large). RR cutoffs were defined as 1–1.22 (small), 1.22–1.86 (moderate), 1.86–3.00 (median), and ≥ 3.00 (large), based on a previous study [61]. To the best of our knowledge, there are no widely accepted guidelines for categorizing RD magnitudes. In practice, researchers often avoid directly synthesizing RDs, opting instead to synthesize relative measures such as OR and RR and then convert them to RD based on specific control risks [62]. Consequently, we did not consider RD in our analysis regarding effect magnitudes. The code for our study is available at https://osf.io/5aq9m/.

## Results

### Characteristics

We included a total of 3,929 MAs, of which 333 had non-convergence with REML by either effect measure. After excluding them, 3,596 MAs were included in our analysis. Table 1 summarizes the characteristics of these MAs, which included data from 25,226 studies (median = 4 studies, IQR = 3 to 8 within MAs). The median total sample size was 723 (IQR = 291 to 1,934), and the median total number of events was 130 (IQR = 46 to 403). Based on the OR with REML and HKSJ (scenario 1), the synthesized results of 1,040 (28.92%) MAs were statistically significant. In the subsequent analysis, we will focus on the MAs that had statistically significant results in the corresponding scenario for each effect measure. For the main analysis, we focused on the empirical distribution of FI based on the scenario of REML with HKSJ (scenario 1), which was presented in the following and Additional file 1. Results for the FE model and the other three scenarios are presented in Additional files 1–5 of the Supplementary Materials.

**Table 1:** Characteristics of the meta-analyses, among the ones with statistically significant synthesized results, statistically nonsignificant synthesized results, and combined ones based on scenario 1 (the REML estimator and the HKSJ method for deriving CIs), with OR as the effect measure

Characteristics	Statistically significant	Statistically nonsignificant	Combined
	**(*N* = 1040)**	**(*N* = 2556)**	**(*N* = 3596)**
**Number of studies, median (IQR)**	7 (4,13)	4 (2, 6)	4 (3, 8)
**Total sample size, median (IQR)**	1282 (524, 3365)	575 (243, 1488)	723 (291, 1934)
**Total number of events, median (IQR)**	268 (96, 801)	98 (36, 288)	130 (46, 403)
**FI, median (IQR)**	5 (2, 14)	5 (2, 9)	5 (2, 10)
**FQ, median (IQR)**	0.0047 (0.0018, 0.0099)	0.0086 (0.0035, 0.0181)	0.0071 (0.0028, 0.0155)

### FI distributions

**Fig. 1:**
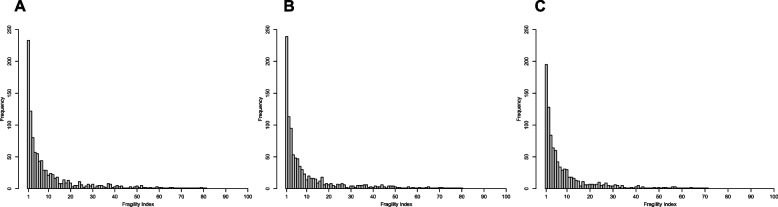
Histogram of the empirical distribution of FI for significant meta-analyses using the REML estimator and the HKSJ method for deriving CIs (scenario 1), with OR (**A**), RR (**B**), and RD (**C**) as the effect measure

We derived empirical FI distributions based on the four scenarios for statistical methods of MAs. In our main results, we used the OR as an effect measure. Figure 1A presents the FI distribution of MAs based on the REML estimator and HKSJ (scenario 1). Based on the results from scenario 1, the median FI was 5 (IQR = 2 to 14). In total, 233 (22.40%) of MAs had an FI of 1, and 547 (52.60%) had an FI of 5 or less.

### Subgroup analyses

**Fig. 2:**
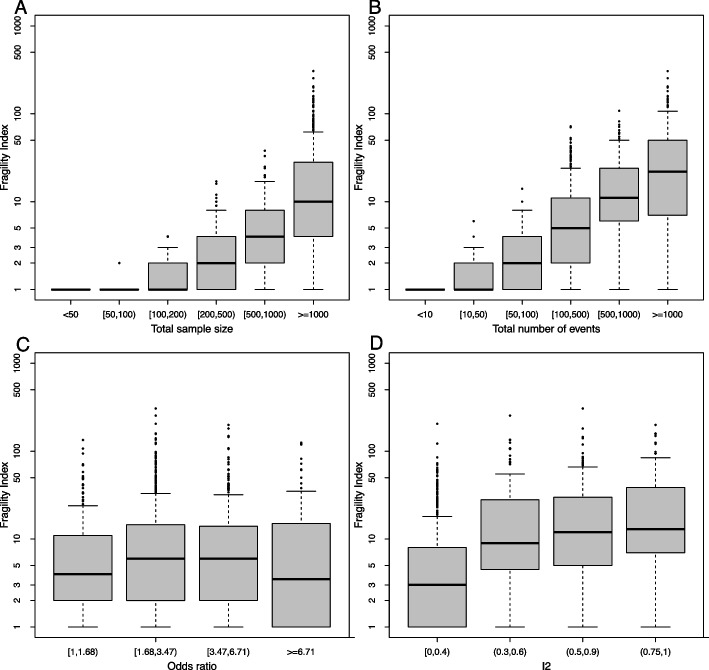
The FI categorized by total sample size (**A**), total number of events (**B**), odds ratio (**C**), and ${I}^{2}$ (**D**) for statistically significant meta-analyses based on scenario 1 (the REML estimator and the HKSJ method for deriving CIs). Total sample size and total number of events correspond to the sum of the sample sizes and the number of events in the trials included in the meta-analyses, respectively. The FI is presented on a logarithmic scale, and the analysis is limited to MAs with FI ≤ 1000

Figure 2 presents the FI of MAs categorized by various factors. In general, the median FI tended to increase with the total number of events. For MAs with a total sample size of less than 1,000, over half had an FI of 5 or lower. The proportion of MAs with an FI of 1 was 100%, 94.74%, and 65.52% for total sample sizes of less than 50, 50–100, and 100–200, respectively. As anticipated, the proportion of MAs with an FI of 5 or less decreased as the total sample size increased.

Specifically, 233 (22.40%) MAs had an FI of 1. We found that some MAs with more than 1000 samples or events could have an FI of 1 (Figs. 2A and B). When categorized by different magnitudes of ORs, MAs with smaller effect sizes tended to be more fragile, but MAs with extremely large ORs (beyond 6.71) could also be fragile (Fig. 2C). This fragility in MAs with very large ORs may be attributed to the substantial uncertainties in the OR estimates, even though the point estimates are far from the null.

Regarding the impact of ${I}^{2}$, MAs with higher ${I}^{2}$ values tended to have larger FIs (Fig. 2D). The heterogeneity of 675 significant MAs might not be important (${I}^{2}$<0.4); 31.56% of them had an FI of 1, and 65.63% had an FI of 5 or less. However, when the MAs had substantial or considerable heterogeneity, only a few (5.53%) had an FI of 1, and about 27% had an FI of 5 or less.

To further investigate the relationship between FI and between-study heterogeneity, we conducted a subgroup analysis based on the number of studies. In the low number of studies group, heterogeneity had a pronounced impact on FI (Additional file 1: Figure S1). We also examined the relationship between FI and the between-study standard deviation $\tau$ categorized by the number of studies (Additional file 1: Figure S2). When the number of studies was small (≤ 14), FI remained relatively stable across different levels of $\tau$. However, for larger numbers of studies (> 14), FI showed a slight increase with greater $\tau$. This modest increase in FI may be attributed to the broad range in the number of studies (from 15 to 148). Additionally, when $\tau$ was at a similar level, FI tended to be higher for MAs with more studies.

### FQ of MAs

The median FQ of significant MAs in scenario 1, based on a *P*-value threshold of 0.05, was 0.0047 (IQR = 0.0018 to 0.0099), indicating that the significance of the results relied on fewer than 5 events per 1,000 participants. Approximately 99.71% of significant MAs had an FQ < 0.05, and 75.58% reported an FQ < 0.01 (Table 2).

**Table 2:** Distribution of meta-analyses (cumulative proportions in parentheses) by fragility index and fragility quotient based on different methods for statistically significant meta-analyses using different effect measures

Model	Effect measure					
	OR, N (%)		RR, N (%)		RD, N (%)	
**FI**	FI = 1	FI ≤ 5	FI = 1	FI ≤ 5	FI = 1	FI ≤ 5
Fixed-effect model	108 (6.64)	420 (25.81)	149 (9.44)	504 (31.94)	154 (9.32)	564 (34.12)
Random-effects model						
Scenario 1 (REML with HKSJ)	233 (22.40)	547 (52.60)	239 (23.55)	548 (53.99)	195 (20.40)	531 (55.54)
Scenario 2 (REML without HKSJ)	152 (11.01)	609 (44.13)	176 (13.19)	615 (46.10)	189 (14.16)	629 (47.12)
Scenario 3 (DL with HKSJ)	239 (23.20)	541 (52.52)	236 (23.62)	540 (54.05)	203 (21.37)	520 (54.74)
Scenario 4 (DL without HKSJ)	164 (11.87)	602 (43.56)	173 (12.91)	606 (45.22)	192 (14.28)	619 (46.02)
**FQ**	FQ < 0.01	FQ < 0.05	FQ < 0.01	FQ < 0.05	FQ < 0.01	FQ < 0.05
Fixed-effect model	533 (32.76)	1291 (79.35)	691(43.79)	1444 (91.51)	715 (43.25)	1376 (83.24)
Random-effects model						
Scenario 1 (REML with HKSJ)	786 (75.58)	1037 (99.71)	781 (76.95)	1009 (99.41)	728 (76.15)	954 (99.79)
Scenario 2 (REML without HKSJ)	811 (58.77)	1337 (96.88)	790 (59.22)	1302 (97.60)	830 (62.17)	1261 (94.46)
Scenario 3 (DL with HKSJ)	763 (74.08)	1027 (99.71)	756 (75.68)	990 (99.10)	721 (75.89)	948 (99.79)
Scenario 4 (DL without HKSJ)	766 (55.43)	1330 (96.24)	762 (56.87)	1301 (97.09)	808 (60.07)	1272 (94.57)

### Comparisons among statistical methods

**Fig. 3:**
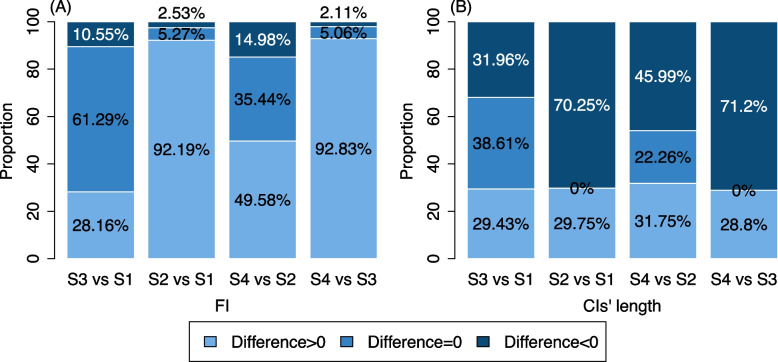
Proportions of paired differences in FI (**A**) and in CI length (**B**) between the two relevant scenarios for the statistically significant meta-analyses using OR as the effect measure. The four scenarios include scenario 1 (REML with HKSJ, abbreviated as S1), scenario 2 (REML without HKSJ, abbreviated as S2), scenario 3 (DL with HKSJ, abbreviated as S3), and scenario 4 (DL without HKSJ, abbreviated as S4). The x-axis labels indicate the paired differences between results from the two scenarios; for example, “S3 vs. S1” in Panel A represents the FI of an individual meta-analysis from scenario 3 minus the corresponding FI from scenario 1

The use of HKSJ appeared to have a greater impact on the fragility of MAs than the choice of heterogeneity variance estimators. Results with HKSJ (e.g., scenario 1 with REML or scenario 3 with DL) were more fragile compared to those without HKSJ, as evidenced by a higher proportion of cases with an FI = 1 and FI ≤ 5 (Table 2). Additionally, we calculated paired differences between two related scenarios for individual MAs with statistically significant results across all scenarios (Fig. 3). Approximately 92% of MAs using heterogeneity estimators without HKSJ had greater FIs than their corresponding scenarios with HKSJ (Fig. 3A).

### Improvement in the FI of an MA

**Fig. 4:**
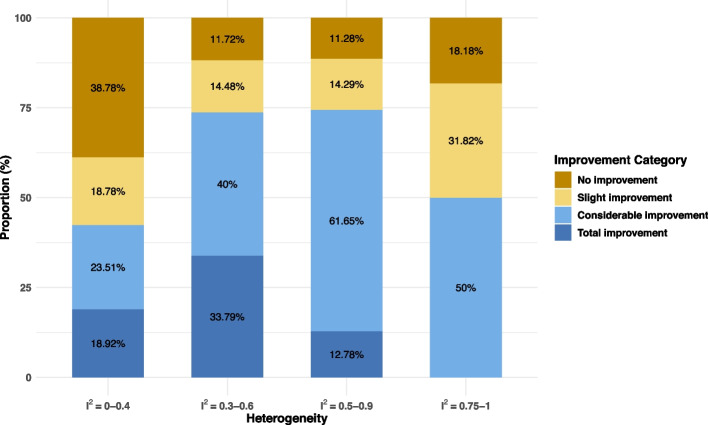
The improvement proportions stratified by ${I}^{2}$ among statistically significant meta-analyses based on scenario 1 (the REML estimator and the HKSJ method for deriving CIs), with OR as the effect measure

We used an improvement proportion to quantify the improvement in fragility by comparing the FI of MAs with the FIs of the individual studies they include. Figure 4 shows the improvement proportions, stratified by ${I}^{2}$. In general, statistically significant MAs with lower heterogeneity tended to show a higher proportion of no or slight improvement. For instance, approximately 37% of significant MAs with small heterogeneity (${I}^{2}$<0.4) showed no improvement (improvement proportion = 0%), whereas over half of those with considerable heterogeneity exhibited substantial improvement (50% < improvement proportion < 100%).

### Effect measures

We also assessed FI using RR and RD to better understand the influence of effect measures on FI. For each effect measure, we used the same framework as in our main analysis.

For RR, 239 (23.55%) of MAs had an FI of 1, and 548 (53.99%) had an FI of 5 or less based on scenario 1 of the REML estimator with HKSJ adjustment (Table 2 and Fig. 1B). Additional file 1: Figure S3 presents the FI of MAs categorized by various factors for scenario 1. The trends observed for total sample size, ${I}^{2}$, and the total number of events were consistent with those for OR. Notably, in the subgroup of medium RR magnitudes, MAs exhibited greater robustness. Across different levels of ${I}^{2}$, higher ${I}^{2}$ values seemed to continue to result in more robust results. Subgroup analyses by the number of studies revealed patterns similar to those observed with OR across different scenarios. They might also be explained by the limitations of using ${I}^{2}$ as the heterogeneity measure (Additional file 1: Figure S4). Additional file 1: Figure S5 illustrates the relationship between FI and the between-study standard deviation $\tau$, categorized by the number of studies across four scenarios. In scenario 1, 1,009 (99.41%) studies had FQs less than 0.05, and 781 (76.95%) of these had FQs less than 0.01 (Table 2).

The paired differences between relevant scenarios for individual MAs are presented in Additional file 1: Figure S6. Approximately 61% of results from heterogeneity estimators without HKSJ adjustments yielded higher FIs than those from the corresponding scenarios with HKSJ adjustments (Supplementary file 3), a proportion similar to that observed with OR. For improvement proportions, Additional file 1: Figure S7 illustrates performance across scenarios stratified by ${I}^{2}$, with an overall pattern consistent with OR.

For RD estimates, 195 (20.40%) MAs had an FI of 1, and 531 (55.54%) had an FI of 5 or less based on scenario 1 (Table 2 and Fig. 1C). Since no widely accepted guidelines exist for categorizing RD magnitudes, FI results are presented only by total sample size and by total sample size combined with ${I}^{2}$ (Additional file 1: Figure S8). Overall, RD showed patterns similar to those of OR and RR across most analyses. Additional file 1: Figure S9 displays the corresponding FI distributions at various ${I}^{2}$ levels. When examining $\tau$, most MAs using RD estimates reported $\tau$ values ranging from 0 to 0.4 (Additional file 1: Figure S10). In all scenarios, over 95% of studies had FQs less than 0.05, while more than 60% had FQs less than 0.01 (Table 2). Paired differences between relevant scenarios for individual MAs are shown in Additional file 1: Figure S11. Finally, Additional file 1: Figure S12 depicts the performance of improvement proportions across different scenarios stratified by ${I}^{2}$, with patterns consistent with those observed for OR and RR.

## Discussion

We have presented empirical distributions of fragility measures of MAs with binary outcomes based on a large collection of Cochrane MAs and extensively explored the association between FI and the characteristics of MAs. While previous empirical investigations of the FI have offered valuable insights, many have been limited to specific clinical domains at the individual trial level, such as ophthalmology, oncology, or cardiology. In contrast, our study evaluates the FI across a broad and diverse set of binary outcome MAs without restriction to any particular medical specialty. To our knowledge, this represents the most comprehensive assessment of the FI in the context of binary outcome MAs to date. Importantly, our study is also the first to systematically compare the FI of MAs with the FIs of the individual studies they include. This added dimension provides a novel perspective on the extent to which meta-analytic synthesis can enhance the robustness of statistical findings.

Based on a recent empirical study on FI in individual RCTs, an FI of 22 may be considered precise and less susceptible to random errors [63]. Applying this threshold to the FIs of Cochrane MAs examined in this study, our primary analysis in scenario 1 (REML with HKSJ) found that the proportions of MAs with FI ≥ 22 were 17.60%, 18.13%, and 14.75% for OR, RR, and RD, respectively. These findings indicate that only a small proportion of MAs may be considered precise.

By comparing fragility based on different statistical methods of MA, we found that HKSJ might impact the fragility of MAs more than the different choices of heterogeneity estimators. HKSJ accounted for more uncertainties in the synthesized results, particularly when the number of studies is small, so they tended to lead to more fragile results. Most MAs had the same FIs/FQs with either REML or DL. For the fragility improvement, MAs with higher heterogeneity tended to have a higher proportion of improvement, meaning that the synthesized results were more robust compared with the majority of the individual studies included in the MAs. Over half of the significant MAs with considerable heterogeneity had considerable improvement.

The observation that MAs with greater heterogeneity appeared to yield more robust results is counterintuitive. This phenomenon may be attributed to the limitations of ${I}^{2}$ as a heterogeneity measure [64]. Specifically, ${I}^{2}$ increases as the total sample size of the MA grows, reducing within-study variances. If between-study variances remain unchanged, ${I}^{2}$ values can approach 100%, potentially overestimating the heterogeneity. In such cases, the narrow CIs resulting from large sample sizes may enhance the robustness of the results.

The FI is correlated with multiple characteristics of MAs. Small-study effects and publication bias may influence the interpretation of fragility measures in MAs. Conceptually, MAs affected by small-study bias could exhibit lower FI values, reflecting that the observed significance may depend disproportionately on smaller, potentially biased studies. However, detecting small-study bias reliably remains challenging, particularly when the number of included studies is limited or when substantial between-study heterogeneity exists. Established methods for identifying publication bias, such as Harbord’s test or Egger’s test, have limited statistical power under these conditions and often yield inconsistent results across regression-based methods [65, 66]. Thus, the true PB is infeasible to capture. Consequently, although the presence of small-study bias is an important consideration when interpreting FI, assessing the association between publication bias and FI robustly is difficult with current methodologies. Future research leveraging improved bias detection techniques may help clarify this relationship. Additionally, future studies linking the FI to formal assessments of evidence certainty, such as GRADE (Grading of Recommendations Assessment, Development and Evaluation) ratings, could provide deeper insights into the relationship between statistical robustness and overall confidence in meta-analytic findings. Moreover, mega-trials may influence the fragility of MAs by contributing disproportionately to the pooled estimates. A very large individual study can dominate the weighting in an MA, substantially narrowing the overall CI and stabilizing the pooled effect estimate. As a result, the statistical significance of the meta-analytic result may become heavily dependent on the findings of a single study, potentially inflating the observed FI and creating a misleading impression of robustness.

This study has some limitations. First, our empirical analysis considered all three effect measures, OR, RR, and RD, regardless of the measures used in the original MAs. The appropriateness of the chosen effect measure may vary on a case-by-case basis. Previous studies have shown that RDs can exhibit greater heterogeneity compared to ORs and RRs [48, 67, 68], which could influence the generalizability of the synthesized results. Additionally, in our analysis of the relationship between effect size magnitudes and FI, there is no widely accepted standard for categorizing the magnitudes of RDs. In practice, researchers often avoid synthesizing RDs directly [33, 62]; instead, they typically synthesize relative measures, such as ORs and RRs, and subsequently convert these to RDs based on a specific control risk. For this reason, our analysis of effect size magnitudes did not apply to RDs.

Second, the extracted database from the Cochrane Library encompassed a wide range of healthcare-related topics. Due to the large sample of MAs analyzed in this study, we were unable to stratify the MAs by specific therapeutic areas or disease topics. This may have contributed to the clinical and contextual variability across MAs. Furthermore, many MAs in our database included only a small number of studies: approximately 37% had five or fewer studies. Extra caution is warranted when selecting heterogeneity measures and comparing fragility improvements for MAs with such limited sample sizes [14, 42, 46, 48].

Third, the fragility improvement metric in this study was based solely on the FI values. Given the correlation between FI and factors such as the number of events, p-value, and effect measures, we recommend that the assessment of fragility improvement in MAs also account for additional considerations. These include clinical insights, study design, and the specific methods employed in the MAs. Furthermore, alternative definitions may be explored to quantify fragility improvement. For instance, one alternative metric could be the proportion of MAs in which the FI of the MA exceeds the sum of the FIs of its individual studies. Different metrics would offer distinct interpretations, potentially reflecting varying conditions for improvement.

## Conclusions

Our findings highlight substantial variability in fragility across MAs and emphasize the influence of heterogeneity, sample size, and statistical methods on the FI. Over half of statistically significant MAs had an FI of five or less, and 22% could lose significance by changing the event status of just one participant. Even at similar levels of between-study heterogeneity, MAs with more included studies tended to have higher FIs, likely due to greater flexibility in redistributing events. Beyond these empirical patterns, our results offer practical benchmarks: researchers can compare the FI of a new MA to our empirical distributions, possibly stratified by factors such as the number of studies, to assess whether its robustness is typical. This contextualization may support more informed interpretations of FI in practice. Further research is needed to refine fragility metrics and better integrate them with clinical relevance and evidence certainty.

## Supplementary Information

Additional file 1: Figure S1. The FI categorized by *I*^2^ in four subgroups based on the number of studies in scenario 1 (the REML estimator and the HKSJ method for deriving CIs), with OR as the effect measure. Figure S2. The FI categorized by the between-study standard deviation *τ* in four subgroups based on the number of studies in scenario 1 (the REML estimator and the HKSJ method for deriving CIs), with OR as the effect measure. Figure S3. The FI categorized by total sample size (A), total number of events (B), relative risk (C), and *I*^2^ (D) for statistically significant meta-analyses based on scenario 1 (the REML estimator and the HKSJ method for deriving CIs), with RR as the effect measure. Figure S4. The FI categorized by *I*^2^ in four subgroups based on the number of studies in scenario 1 (the REML estimator and the HKSJ method for deriving CIs), with RR as the effect measure. Figure S5. The FI categorized by the between-study standard deviation *τ* in four subgroups based on the number of studies in scenario 1 (the REML estimator and the HKSJ method for deriving CIs), with RR as the effect measure. Figure S6. Proportions of paired differences in FI (A) and in CI length (B) between the two relevant scenarios for the statistically significant meta-analyses using RR as the effect measure. Figure S7. The improvement proportions stratified by *I*^2^ among statistically significant meta-analyses based on scenario 1 (the REML estimator and the HKSJ method for deriving CIs), with RR as the effect measure. Figure S8. The FI categorized by total sample size (A), total number of events (B), and *I*^2^ (C) for statistically significant meta-analyses based on scenario 1 (the REML estimator and the HKSJ method for deriving CIs), with RD as the effect measure. Figure S9. The FI categorized by *I*^2^ in four subgroups based on the number of studies in scenario 1 (the REML estimator and the HKSJ method for deriving CIs), with RD as the effect measure. Figure S10. The FI categorized by the between-study standard deviation *τ* in four subgroups based on the number of studies in scenario 1 (the REML estimator and the HKSJ method for deriving CIs), with RD as the effect measure. Figure S11. Proportions of paired differences in FI (A) and in CI length (B) between the two relevant scenarios for the statistically significant meta-analyses using RD as the effect measure. Figure S12. The improvement proportions stratified by *I*^2^ among statistically significant meta-analyses based on scenario 1 (the REML estimator and the HKSJ method for deriving CIs), with RD as the effect measure.

Additional file 2: Results for meta-analyses using the REML estimator and the conventional normality-based method for deriving CIs (scenario 2).

Additional file 3: Results for meta-analyses using the DL estimator and the HKSJ method for deriving CIs (scenario 3).

Additional file 4: Results for meta-analyses using the DL estimator and the conventional normality-based method for deriving CIs (scenario 4).

Additional file 5: Results for meta-analyses using the fixed-effect model.

## Data Availability

No datasets were generated or analysed during the current study.

## Abbreviations

CI: Confidence interval
DL: DerSimonian–Laird
FE: Fixed-effect
FI: Fragility index
FQ: Fragility quotient
GRADE: Grading of Recommendations Assessment, Development and Evaluation
HKSJ: Hartung–Knapp–Sidik–Jonkman
MA: Meta-analysis
OR: Odds ratio
RCT: Randomized controlled trial
RD: Risk difference
RE: Random-effects
REML: Restricted maximum-likelihood
RR: Relative risk
