# Supplementary material for: Evaluating the properties of the fragility index of meta-analyses
Source: BMC Med Res Methodol. 2025 Sep 25;25:212. doi: 10.1186/s12874-025-02648-5 (PMC12465983; doi:10.1186/s12874-025-02648-5)
Supplement: Supplementary file 1 — Additional file 1: Figure S1. The FI categorized by I2 in four subgroups based on the number of studies in scenario 1 (the REML estimator and the HKSJ method for deriving CIs), with OR as the effect measure. Figure S2. The FI categorized by the between-study standard deviation τ in four subgroups based on the number of studies in scenario 1 (the REML estimator and the HKSJ method for deriving CIs), with OR as the effect measure. Figure S3. The FI categorized by total sample size (A), total number of events (B), relative risk (C), and I2 (D) for statistically significant meta-analyses based on scenario 1 (the REML estimator and the HKSJ method for deriving CIs), with RR as the effect measure. Figure S4. The FI categorized by I2 in four subgroups based on the number of studies in scenario 1 (the REML estimator and the HKSJ method for deriving CIs), with RR as the effect measure. Figure S5. The FI categorized by the between-study standard deviation τ in four subgroups based on the number of studies in scenario 1 (the REML estimator and the HKSJ method for deriving CIs), with RR as the effect measure. Figure S6. Proportions of paired differences in FI (A) and in CI length (B) between the two relevant scenarios for the statistically significant meta-analyses using RR as the effect measure. Figure S7. The improvement proportions stratified by I2 among statistically significant meta-analyses based on scenario 1 (the REML estimator and the HKSJ method for deriving CIs), with RR as the effect measure. Figure S8. The FI categorized by total sample size (A), total number of events (B), and I2 (C) for statistically significant meta-analyses based on scenario 1 (the REML estimator and the HKSJ method for deriving CIs), with RD as the effect measure. Figure S9. The FI categorized by I2 in four subgroups based on the number of studies in scenario 1 (the REML estimator and the HKSJ method for deriving CIs), with RD as the effect measure. Figure S10. The FI categorized by the betwee [file 12874_2025_2648_MOESM1_ESM.pdf]

Additional File 1 for
“Evaluating the properties of the fragility index in meta-analyses”

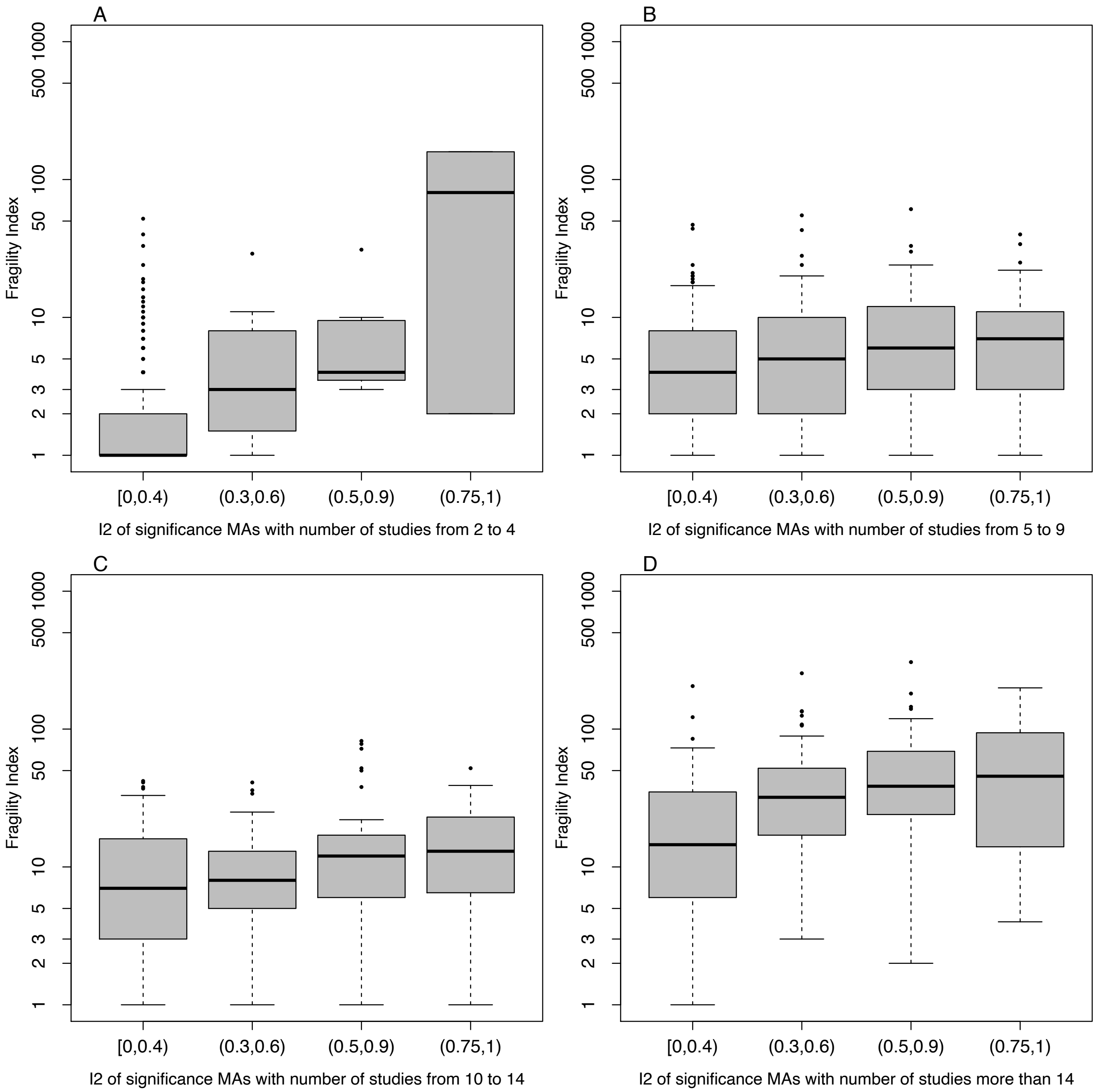

Figure S1. The FI categorized by I^2 in four subgroups based on the number of studies in scenario 1 (the REML estimator and the HKSJ method for deriving CIs), with OR as the effect measure.

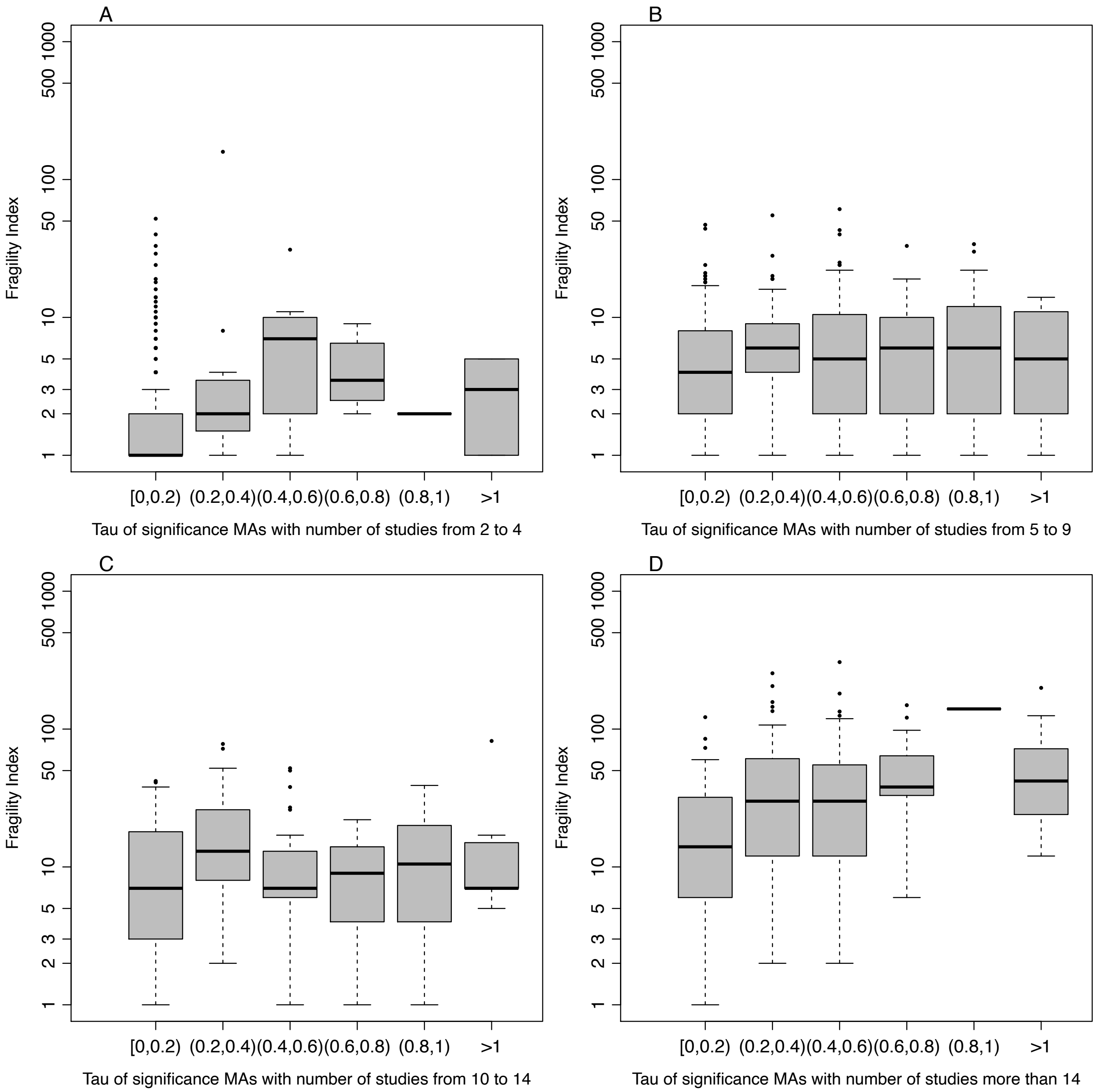

Figure S2. The FI categorized by the between-study standard deviation τ in four subgroups based on the number of studies in scenario 1 (the REML estimator and the HKSJ method for deriving CIs), with OR as the effect measure.

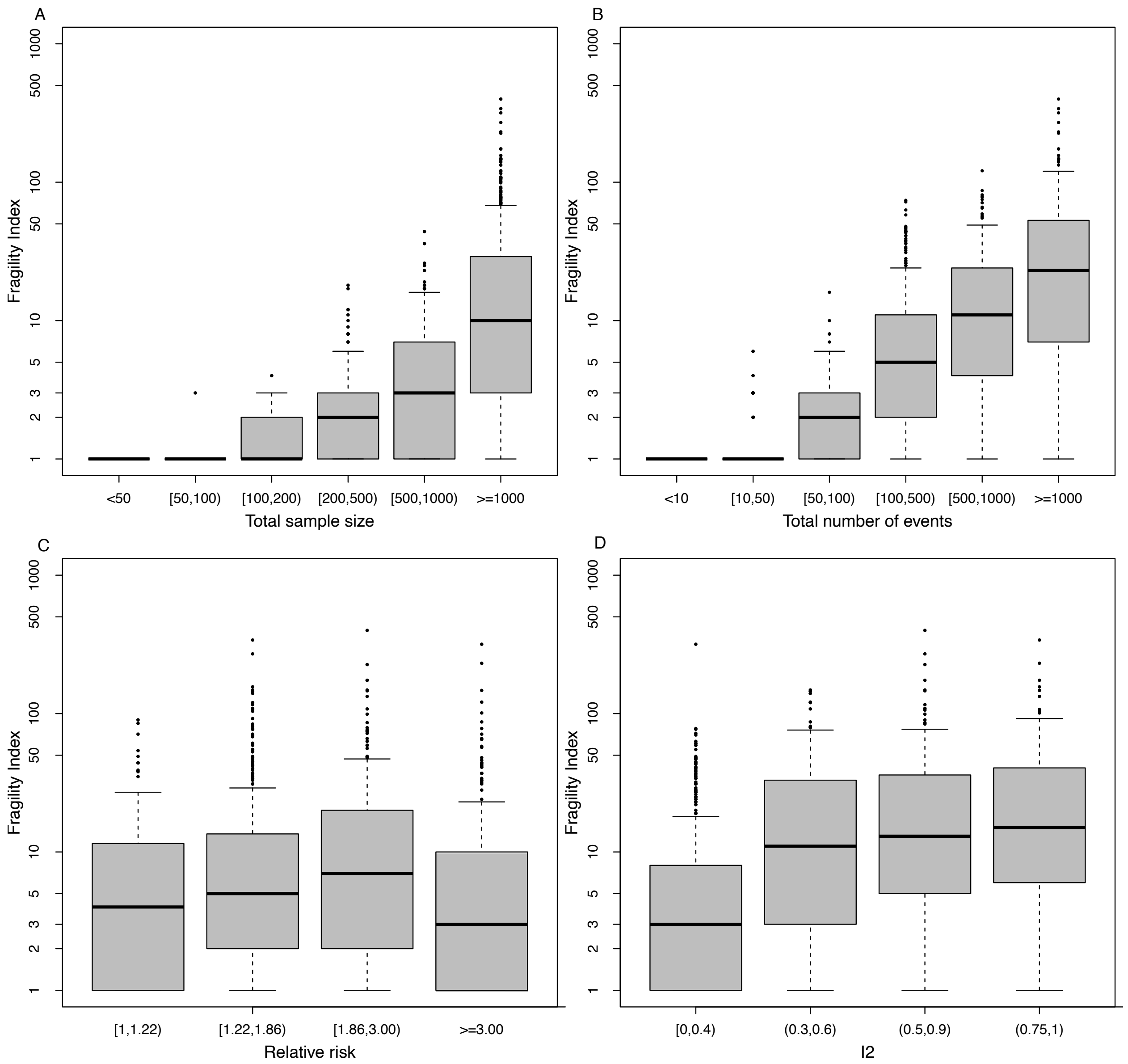

Figure S3. The FI categorized by total sample size (A), total number of events (B), relative risk (C), and I^2 (D) for statistically significant meta-analyses based on scenario 1 (the REML estimator and the HKSJ method for deriving CIs), with RR as the effect measure. Total sample size and total number of events correspond to the sum of the sample sizes and the number of events in the trials included in the meta-analyses, respectively. The FI is presented on a logarithmic scale, and the analysis is limited to MAs with FI≤1000.

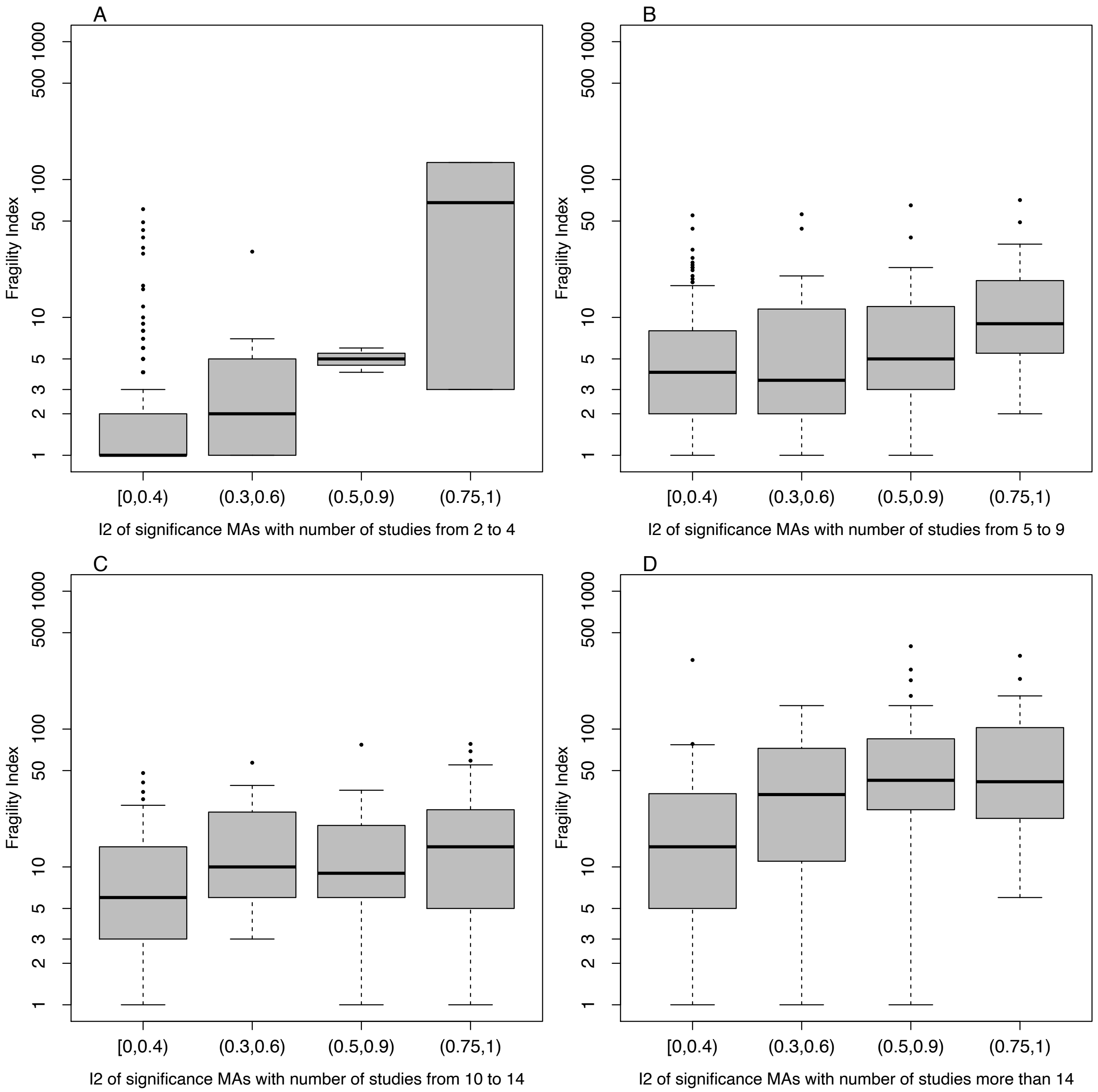

Figure S4. The FI categorized by I^2 in four subgroups based on the number of studies in scenario 1 (the REML estimator and the HKSJ method for deriving CIs), with RR as the effect measure.

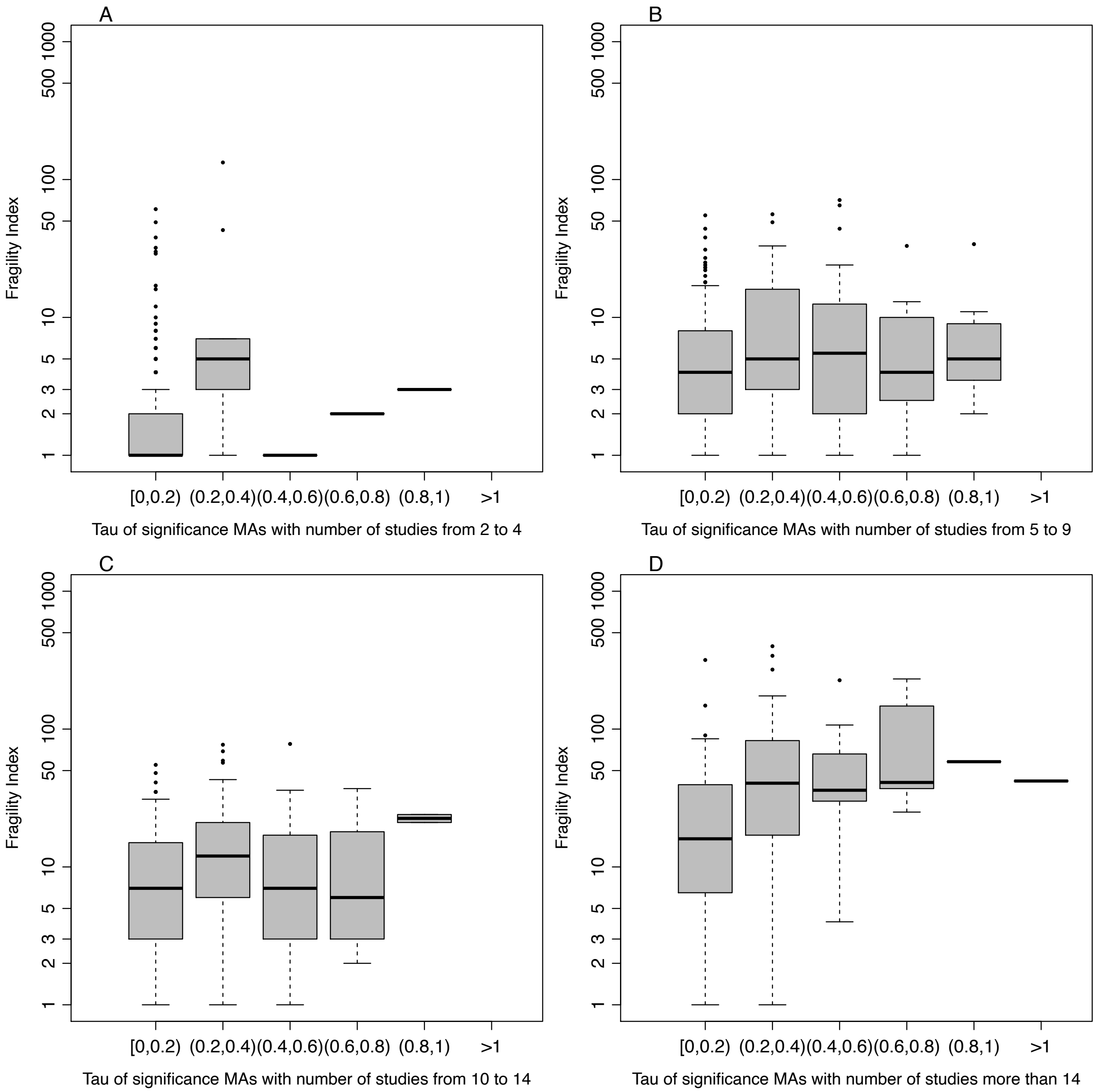

Figure S5. The FI categorized by the between-study standard deviation τ in four subgroups based on the number of studies in scenario 1 (the REML estimator and the HKSJ method for deriving CIs), with RR as the effect measure.

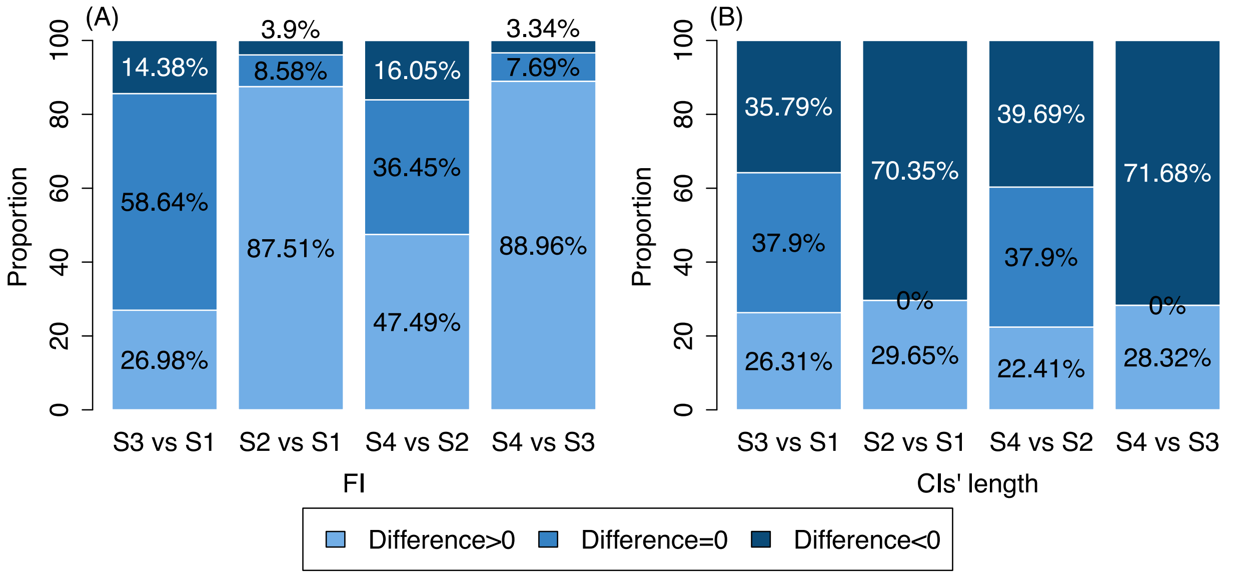

Figure S6. Proportions of paired differences in FI (A) and in CI length (B) between the two relevant scenarios for the statistically significant meta-analyses using RR as the effect measure. The four scenarios include scenario 1 (REML with HKSJ, abbreviated as S1), scenario 2 (REML without HKSJ, abbreviated as S2), scenario 3 (DL with HKSJ, abbreviated as S3), and scenario 4 (DL without HKSJ, abbreviated as S4). The x-axis labels indicate the paired differences between results from the two scenarios; for example, “S3 vs. S1” in Panel A represents the FI of an individual meta-analysis from scenario 3 minus the corresponding FI from scenario 1.

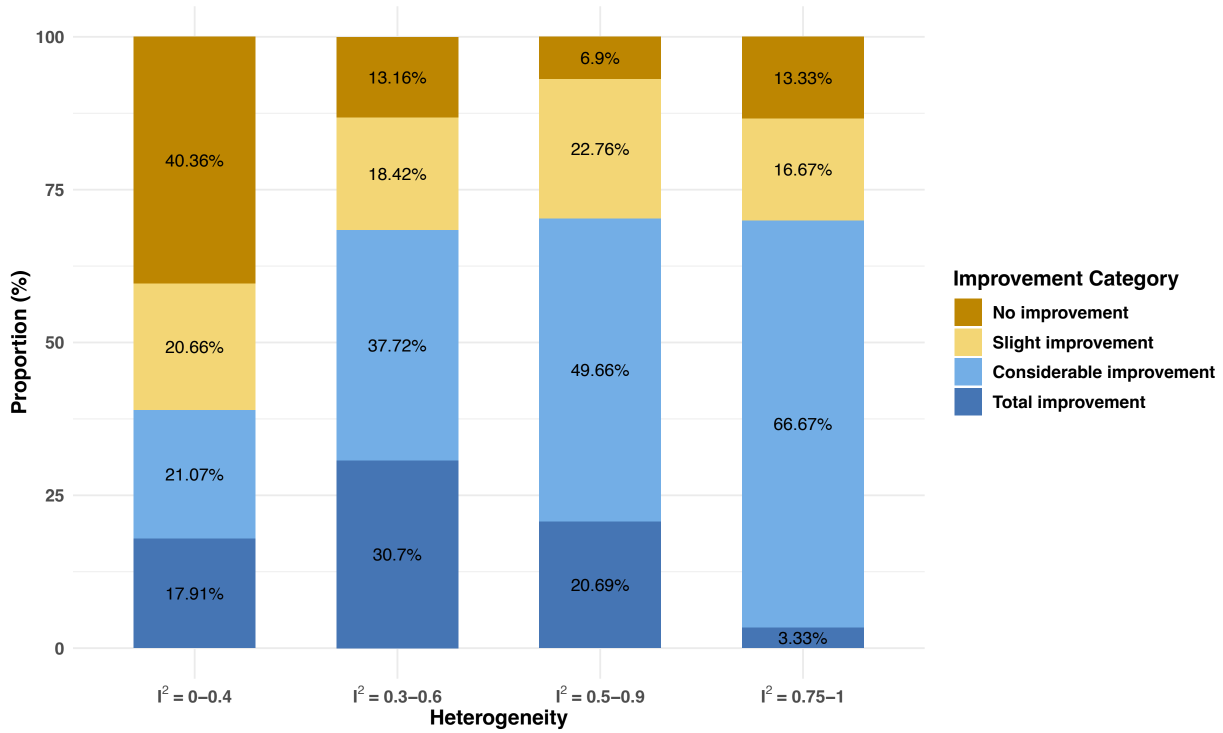

Figure S7. The improvement proportions stratified by I^2 among statistically significant meta-analyses based on scenario 1 (the REML estimator and the HKSJ method for deriving CIs), with RR as the effect measure.

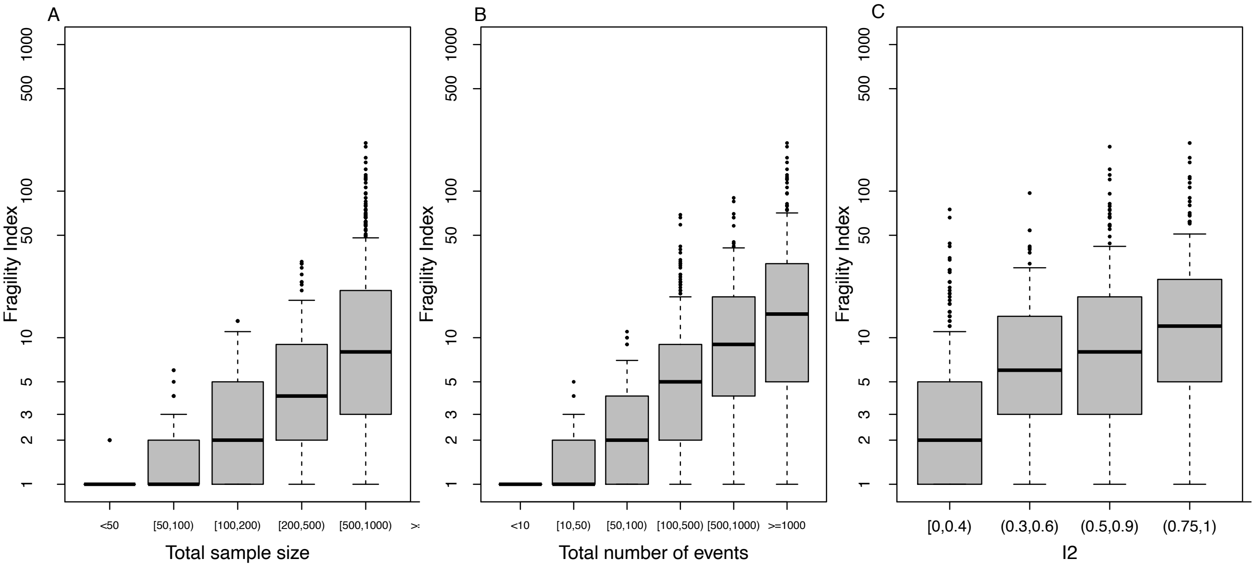

Figure S8. The FI categorized by total sample size (A), total number of events (B), and I^2 (C) for statistically significant meta-analyses based on scenario 1 (the REML estimator and the HKSJ method for deriving CIs), with RD as the effect measure. Total sample size and total number of events correspond to the sum of the sample sizes and the number of events in the trials included in the meta-analyses, respectively. The FI is presented on a logarithmic scale, and the analysis is limited to MAs with FI≤1000.

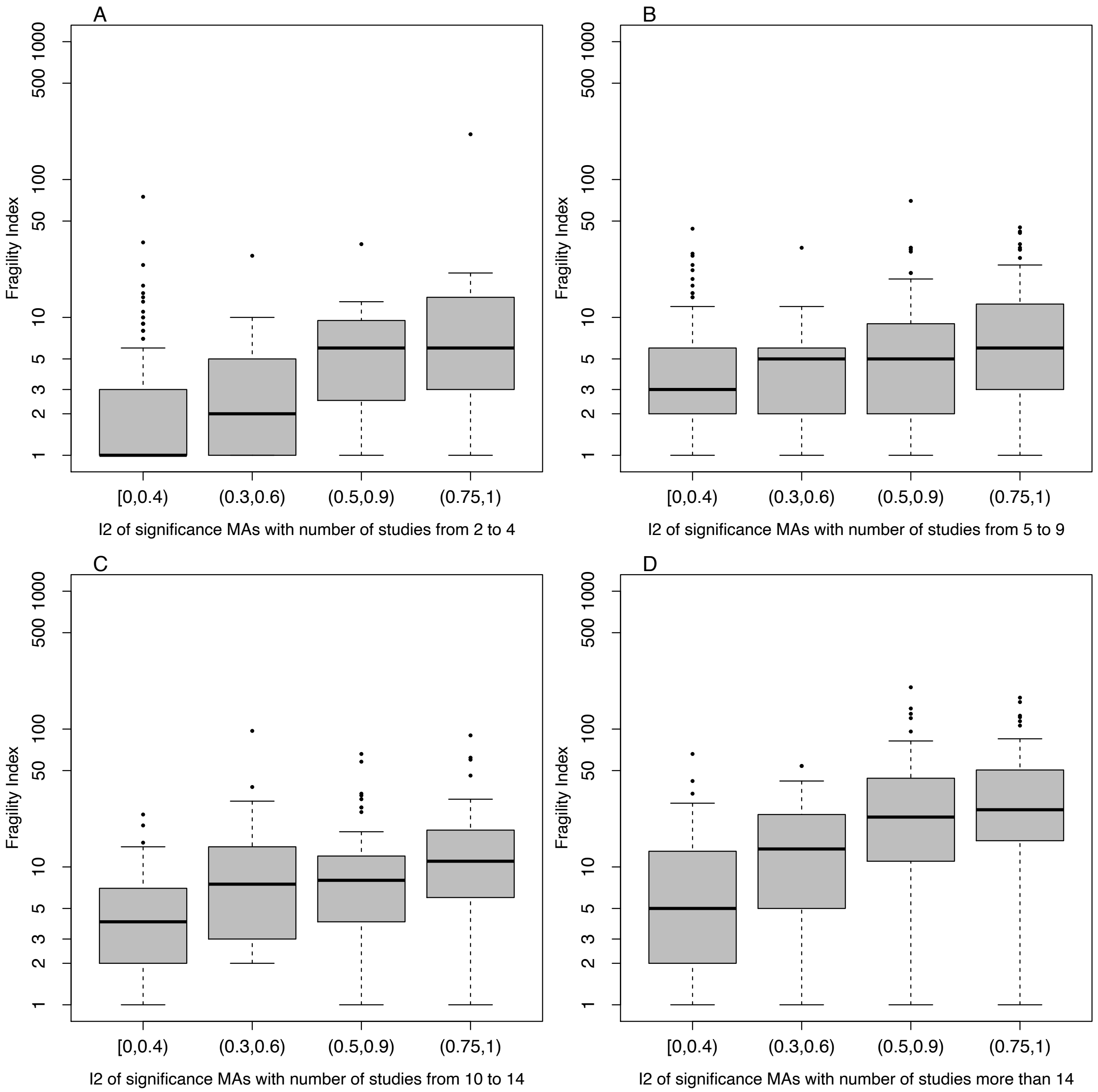

Figure S9. The FI categorized by I^2 in four subgroups based on the number of studies in scenario 1 (the REML estimator and the HKSJ method for deriving CIs), with RD as the effect measure.

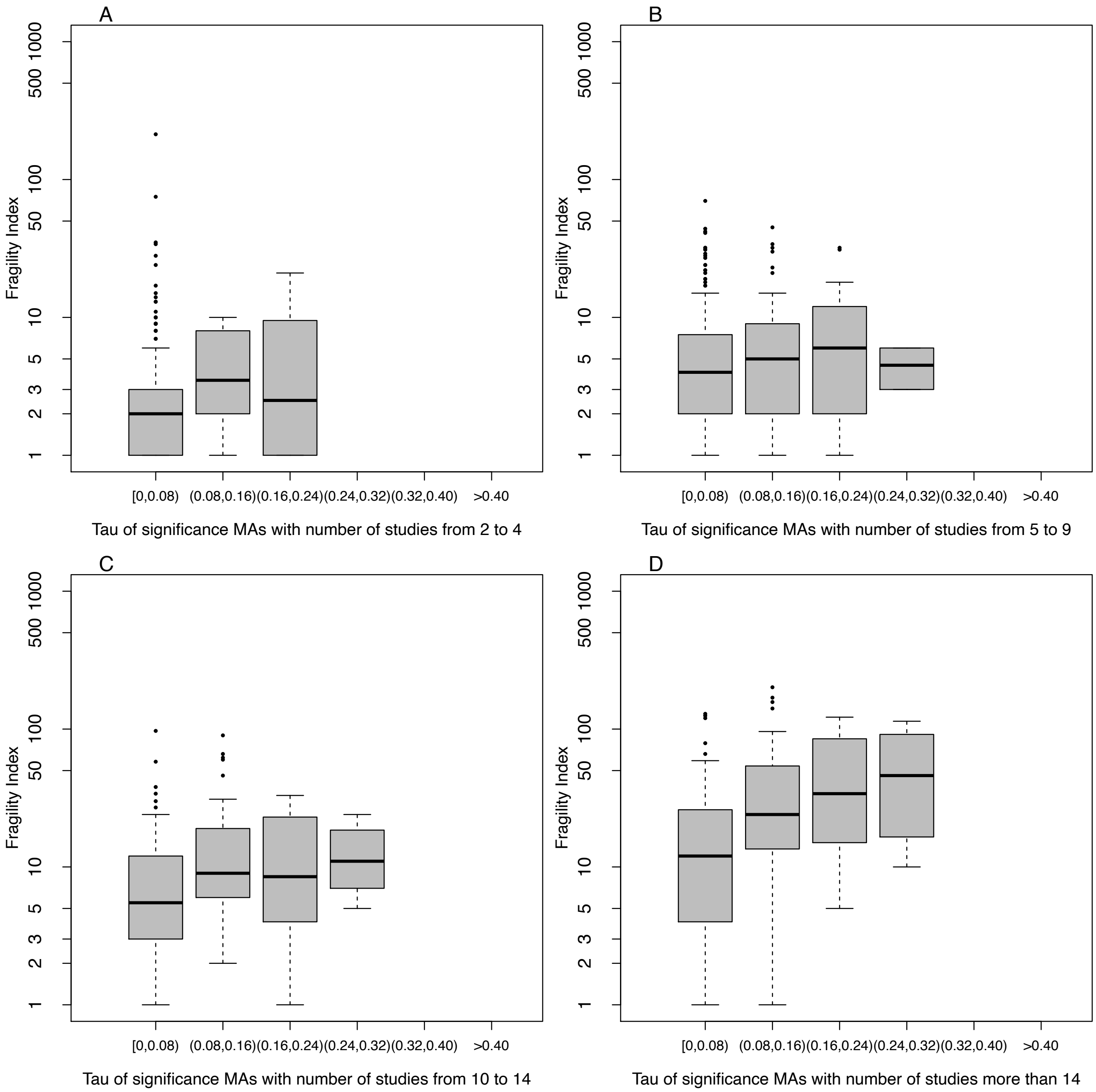

Figure S10. The FI categorized by the between-study standard deviation τ in four subgroups based on the number of studies in scenario 1 (the REML estimator and the HKSJ method for deriving CIs), with RD as the effect measure.

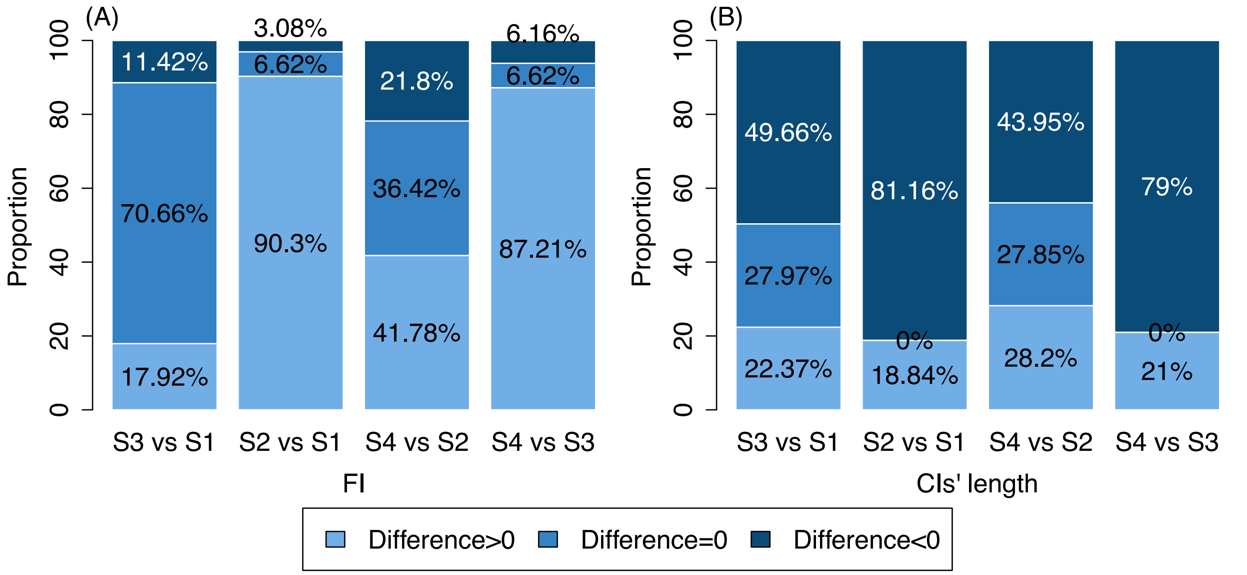

Figure S11. Proportions of paired differences in FI (A) and in CI length (B) between the two relevant scenarios for the statistically significant meta-analyses using RD as the effect measure. The four scenarios include scenario 1 (REML with HKSJ, abbreviated as S1), scenario 2 (REML without HKSJ, abbreviated as S2), scenario 3 (DL with HKSJ, abbreviated as S3), and scenario 4 (DL without HKSJ, abbreviated as S4). The x-axis labels indicate the paired differences between results from the two scenarios; for example, “S3 vs. S1” in Panel A represents the FI of an individual meta-analysis from scenario 3 minus the corresponding FI from scenario 1.

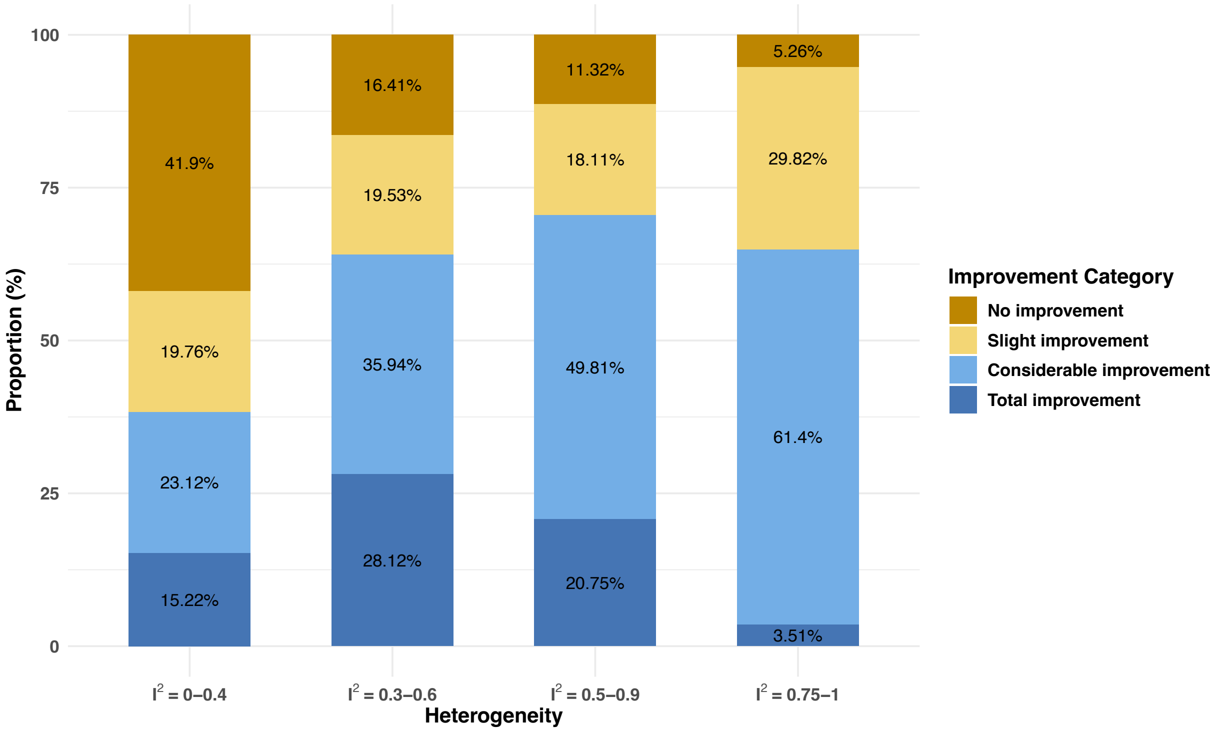

Figure S12. The improvement proportions stratified by I^2 among statistically significant meta-analyses based on scenario 1 (the REML estimator and the HKSJ method for deriving CIs), with RD as the effect measure.
