# Supplementary material for: Evaluating the properties of the fragility index of meta-analyses
Source: BMC Med Res Methodol. 2025 Sep 25;25:212. doi: 10.1186/s12874-025-02648-5 (PMC12465983; doi:10.1186/s12874-025-02648-5)
Supplement: Supplementary file 2 — Additional file 2: Results for meta-analyses using the REML estimator and the conventional normality-based method for deriving CIs (scenario 2). [file 12874_2025_2648_MOESM2_ESM.pdf]

Additional File 2 for
“Evaluating the properties of the fragility index in meta-analyses”

Results for meta-analyses using the REML estimator and the conventional normality-based method for deriving CIs (scenario 2)

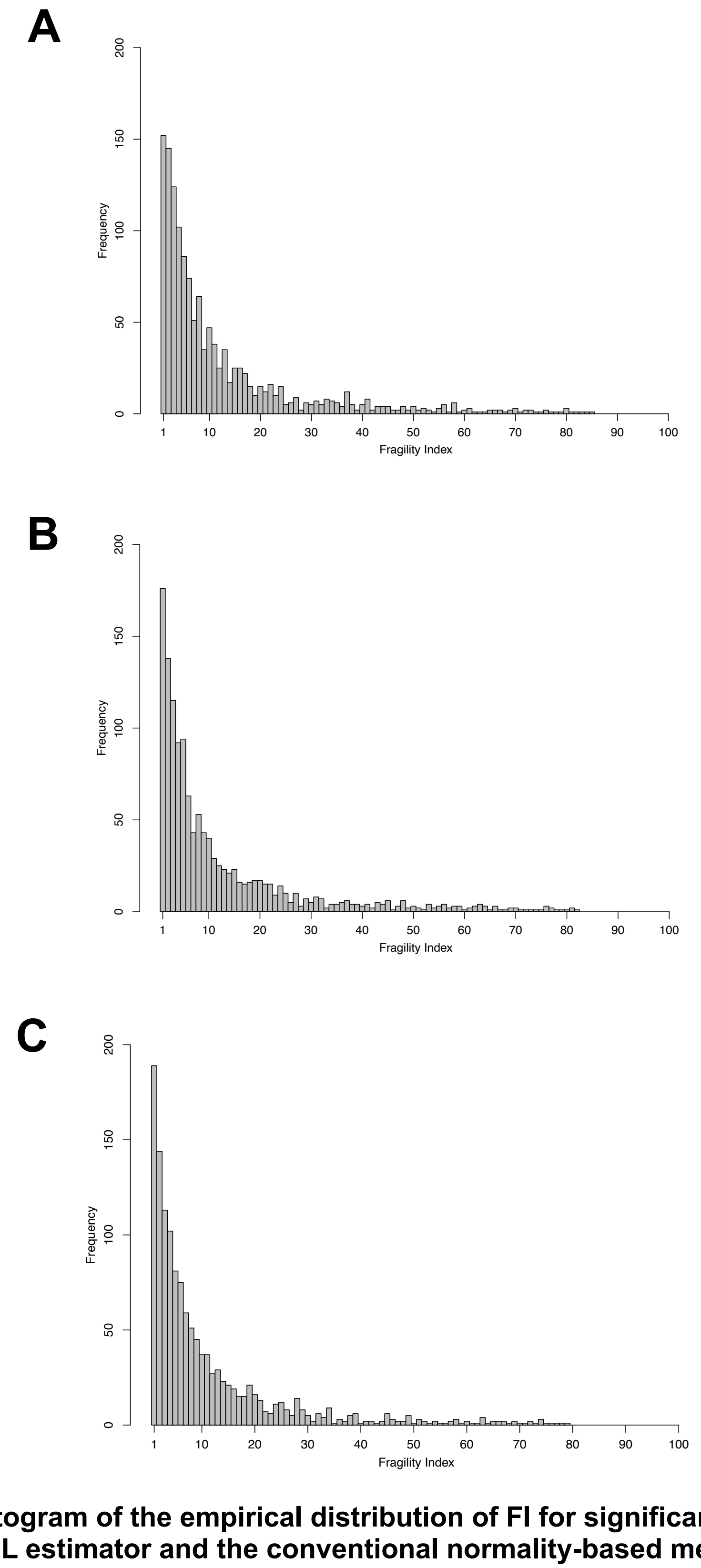

Figure S1. Histogram of the empirical distribution of FI for significant meta-analyses using the REML estimator and the conventional normality-based method for deriving CIs (scenario 2), with OR (A), RR (B), and RD (C) as the effect measure.

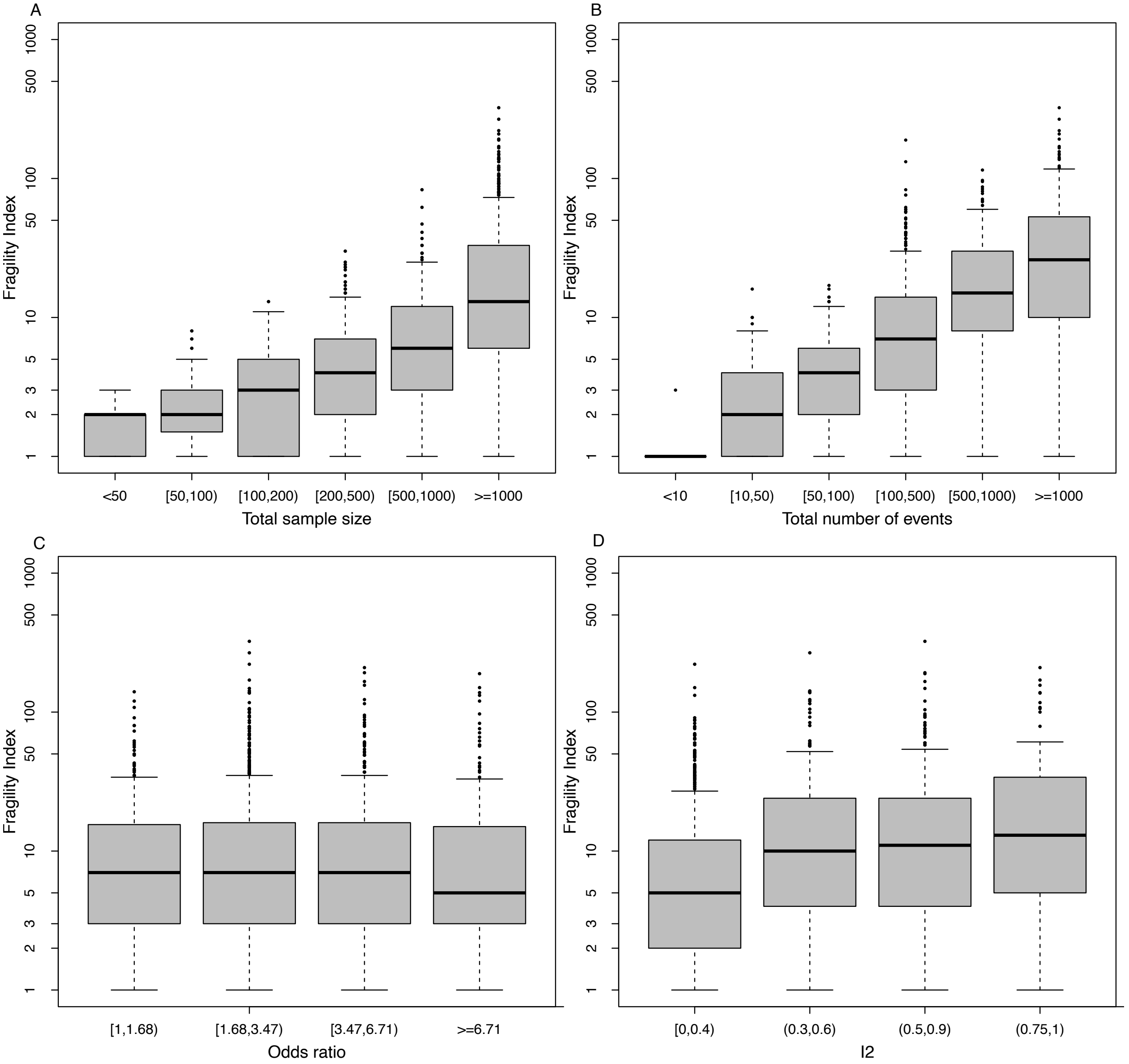

Figure S2. The FI categorized by total sample size (A), total number of events (B), odds ratio (C), and I^2 (D) for statistically significant meta-analyses based on scenario 2 (the REML estimator and the conventional normality-based method for deriving CIs), with OR as the effect measure. Total sample size and total number of events correspond to the sum of the sample sizes and the number of events in the trials included in the meta-analyses, respectively. The FI is presented on a logarithmic scale, and the analysis is limited to MAs with FI≤1000.

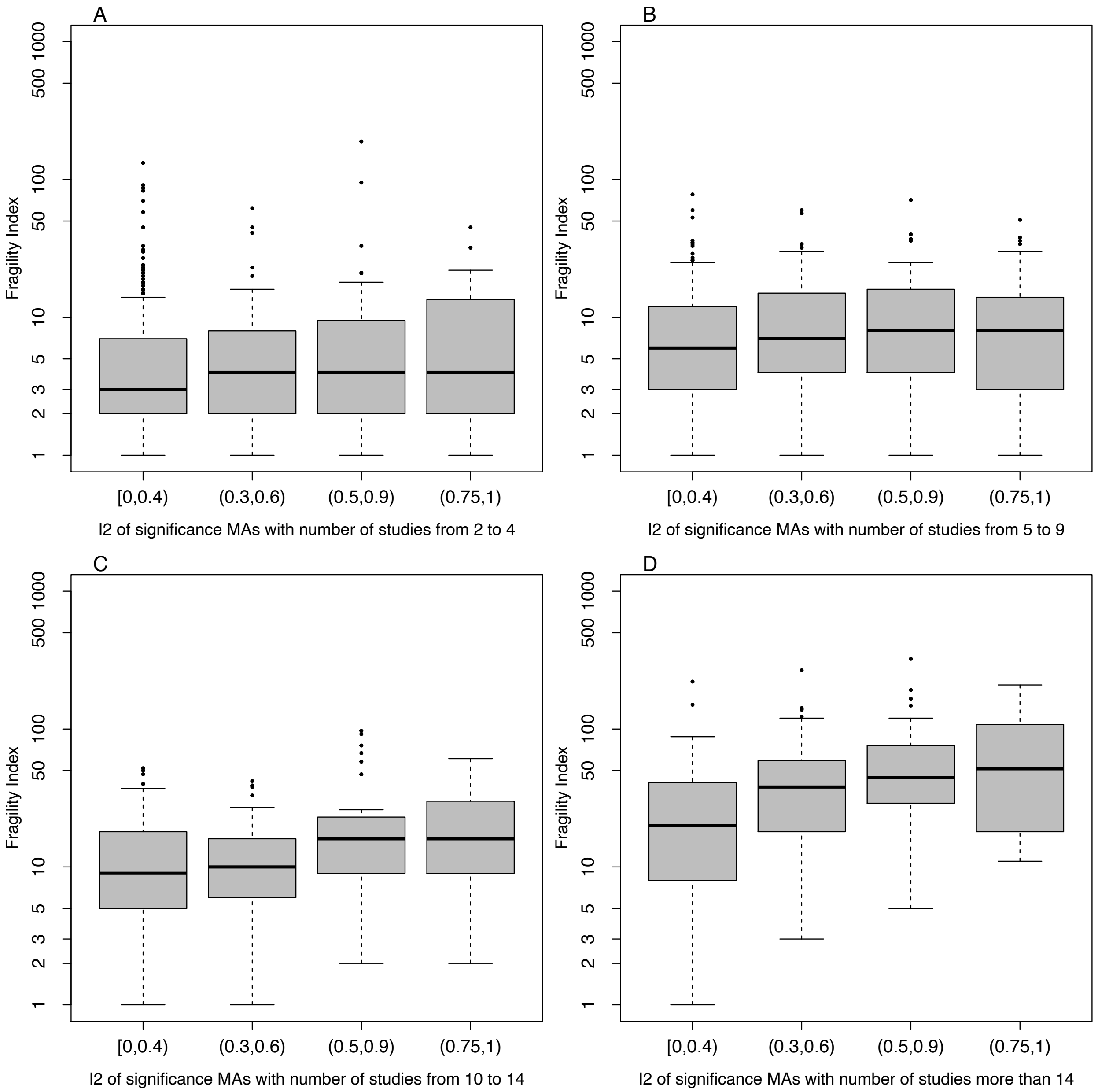

Figure S3. The FI categorized by I^2 in four subgroups based on the number of studies in scenario 2 (the REML estimator and the conventional normality-based method for deriving CIs), with OR as the effect measure.

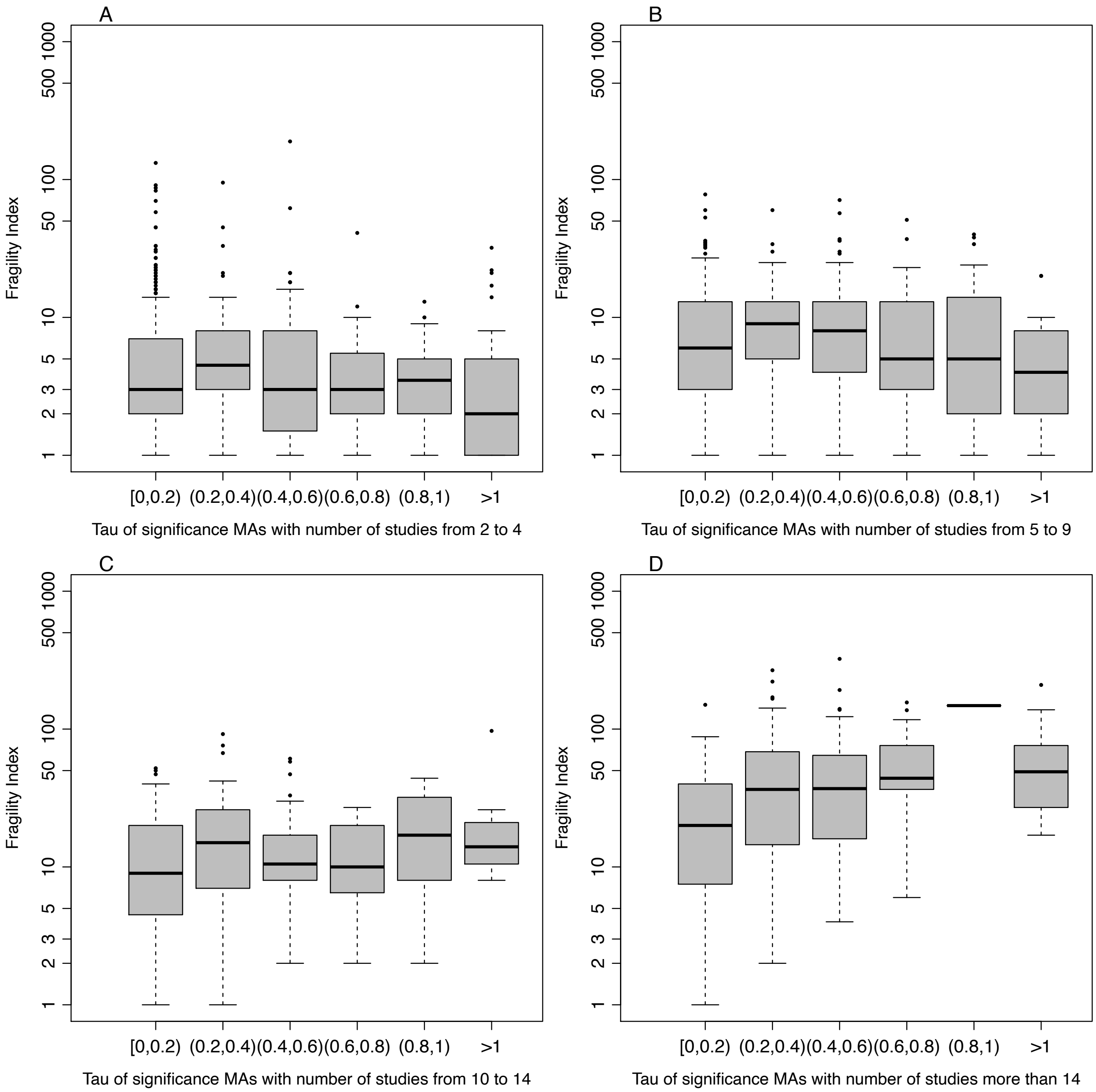

Figure S4. The FI categorized by the between-study standard deviation τ in four subgroups based on the number of studies in scenario 2 (the REML estimator and the conventional normality-based method for deriving CIs), with OR as the effect measure.

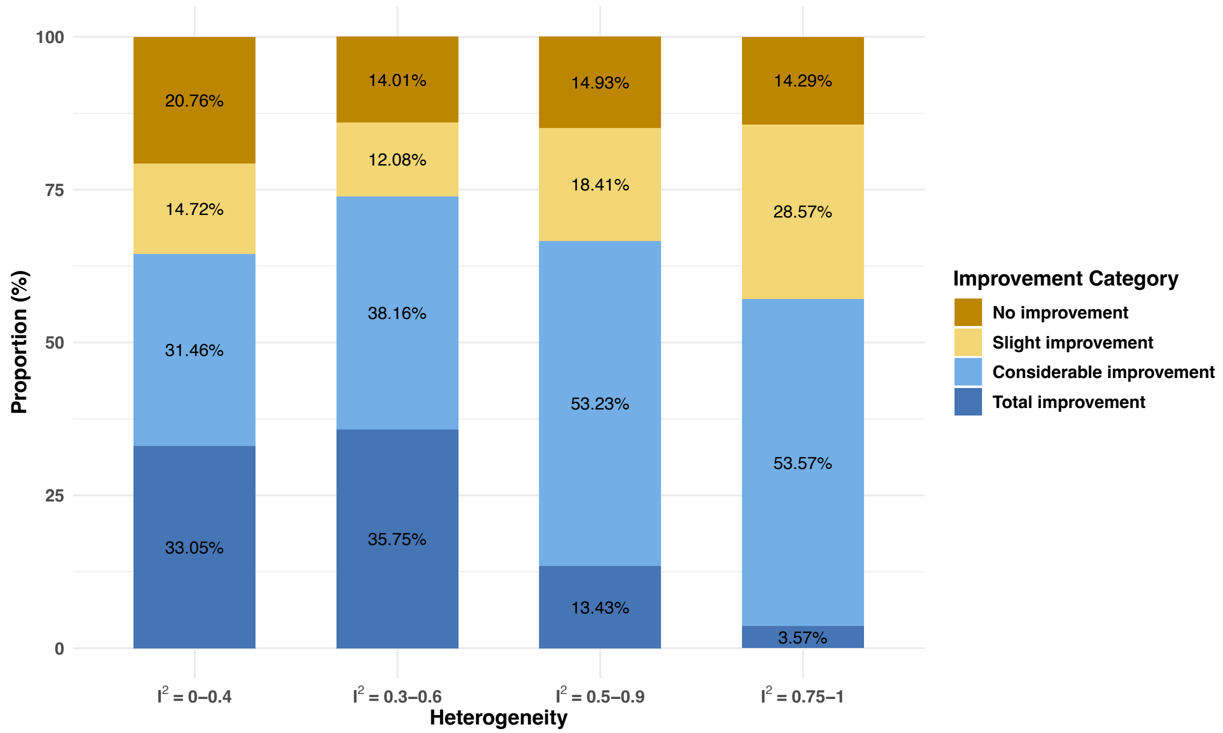

Figure S5. The improvement proportions stratified by I^2 among statistically significant meta-analyses based on scenario 2 (the REML estimator and the conventional normality-based method for deriving CIs), with OR as the effect measure.

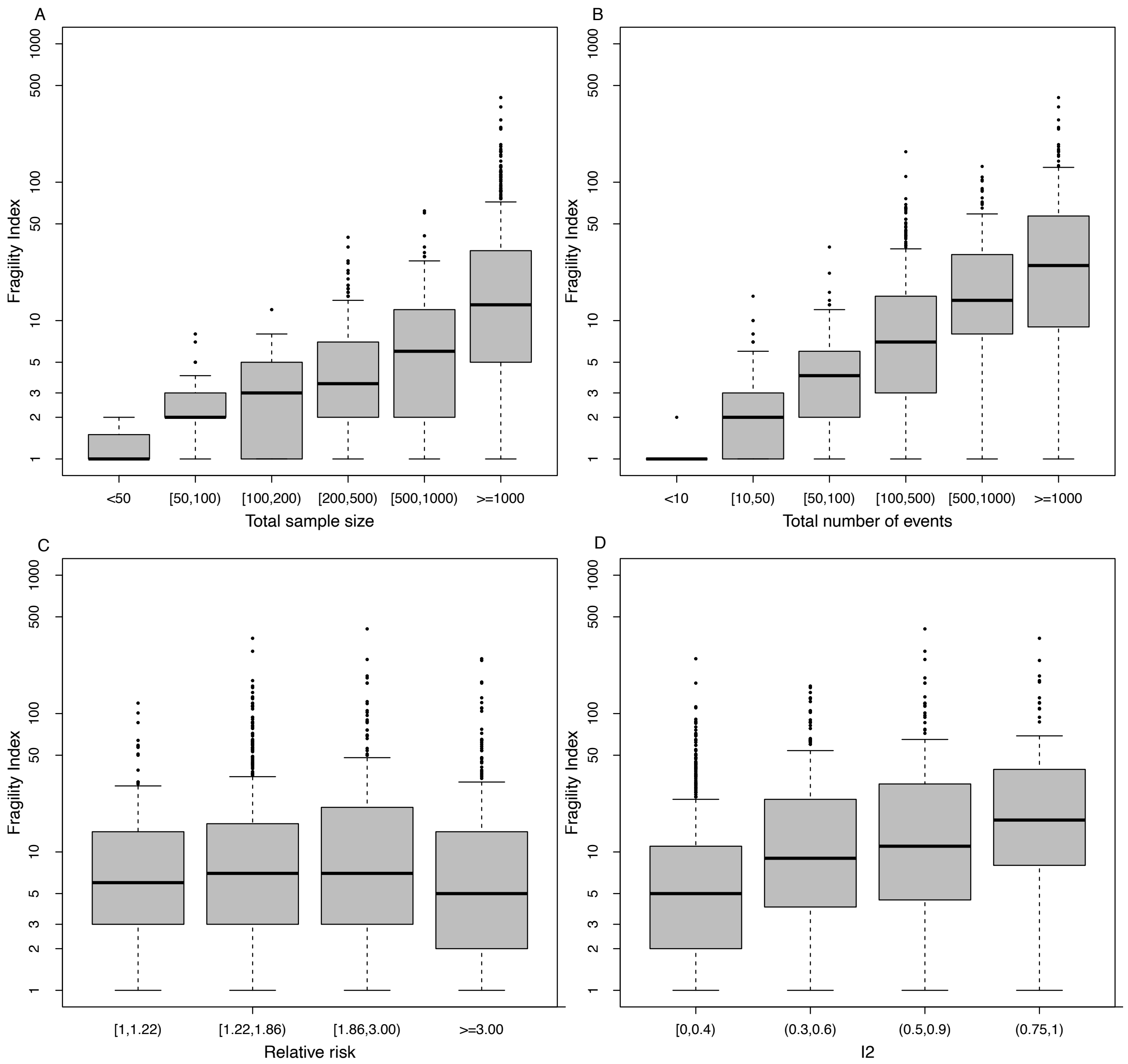

Figure S6. The FI categorized by total sample size (A), total number of events (B), relative risk (C), and I^2 (D) for statistically significant meta-analyses based on scenario 2 (the REML estimator and the conventional normality-based method for deriving CIs), with RR as the effect measure. Total sample size and total number of events correspond to the sum of the sample sizes and the number of events in the trials included in the meta-analyses, respectively. The FI is presented on a logarithmic scale, and the analysis is limited to MAs with FI≤1000.

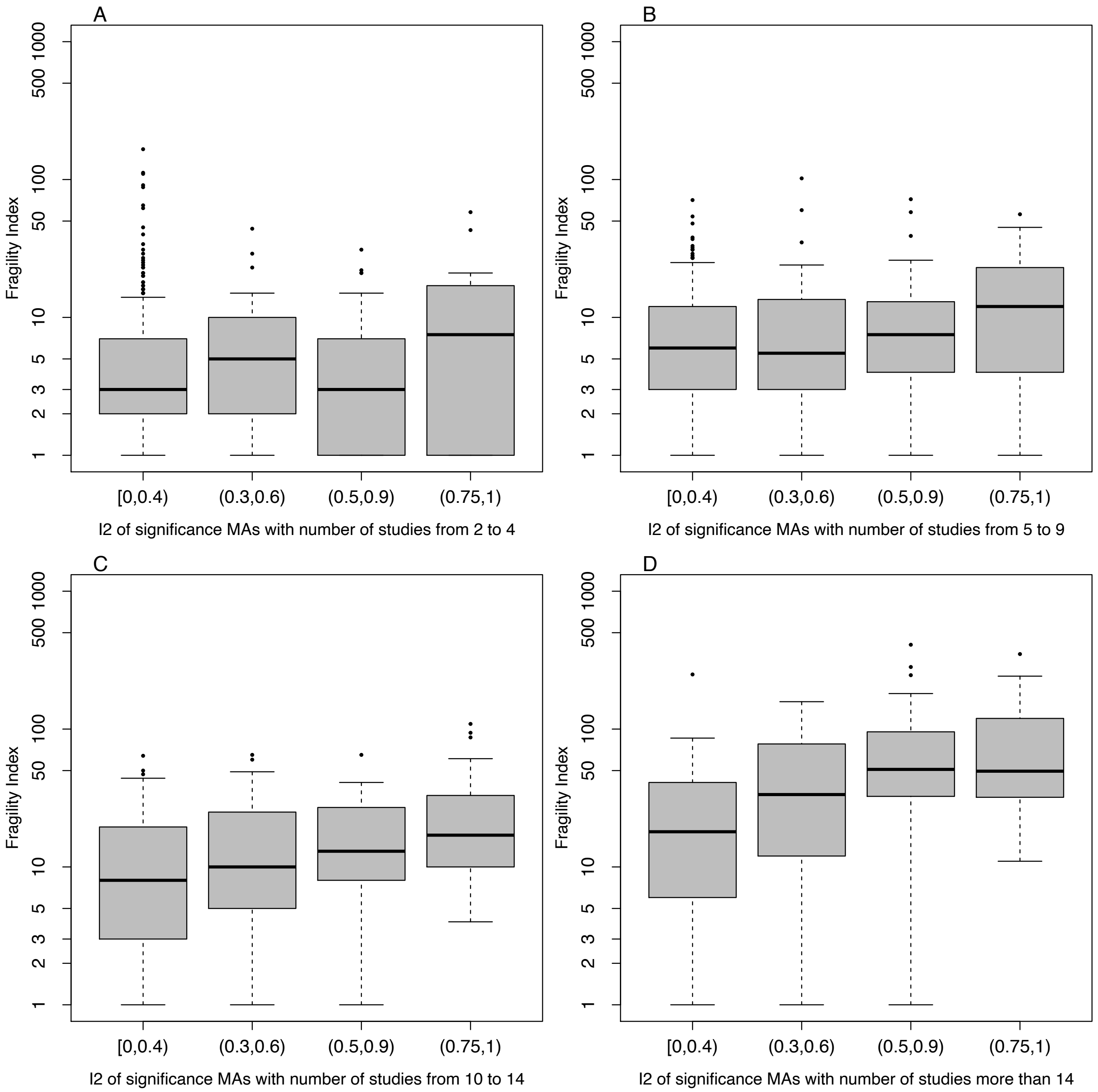

Figure S7. The FI categorized by I^2 in four subgroups based on the number of studies in scenario 2 (the REML estimator and the conventional normality-based method for deriving CIs), with RR as the effect measure.

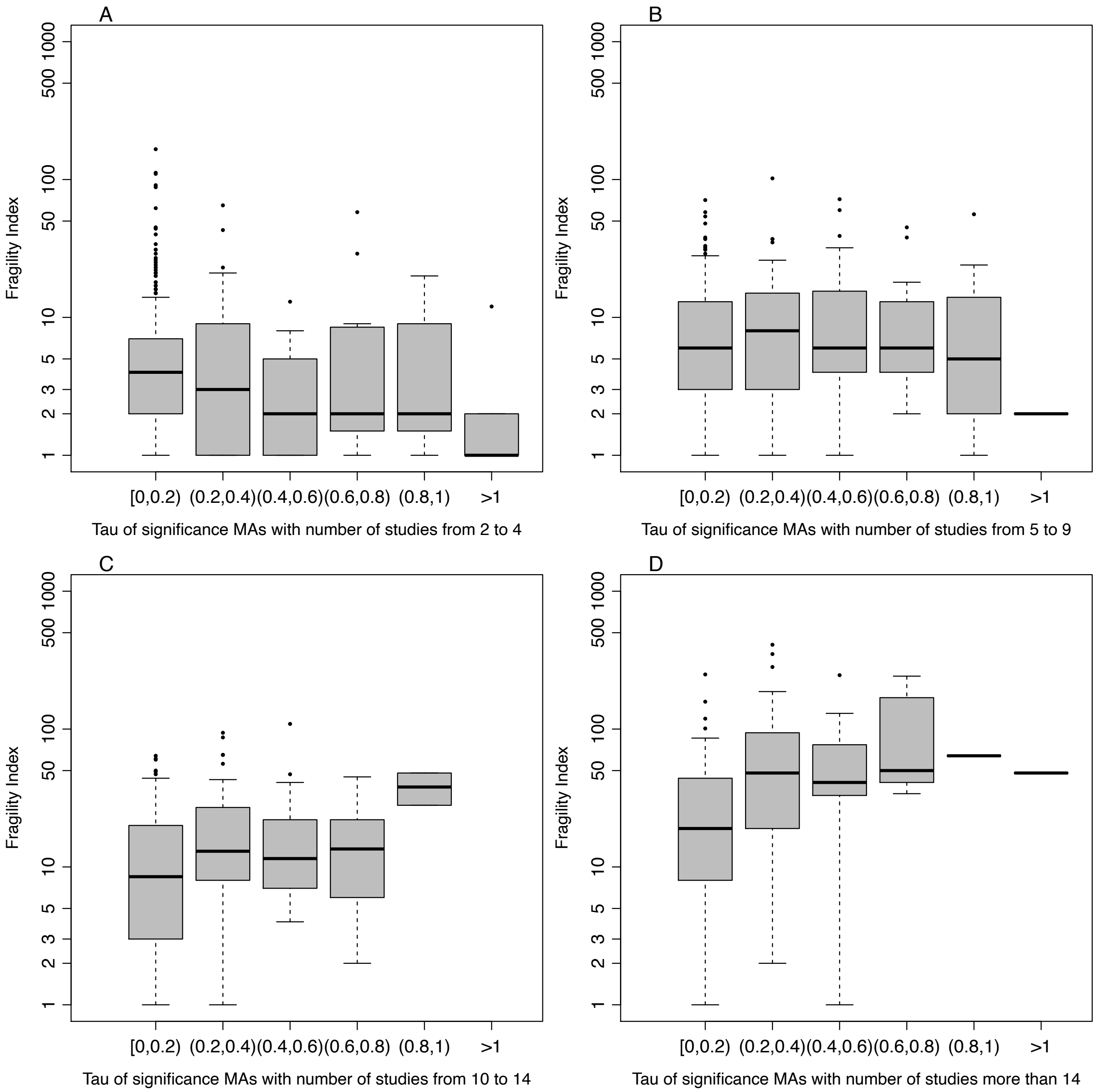

Figure S8. The FI categorized by the between-study standard deviation τ in four subgroups based on the number of studies in scenario 2 (the REML estimator and the conventional normality-based method for deriving CIs), with RR as the effect measure.

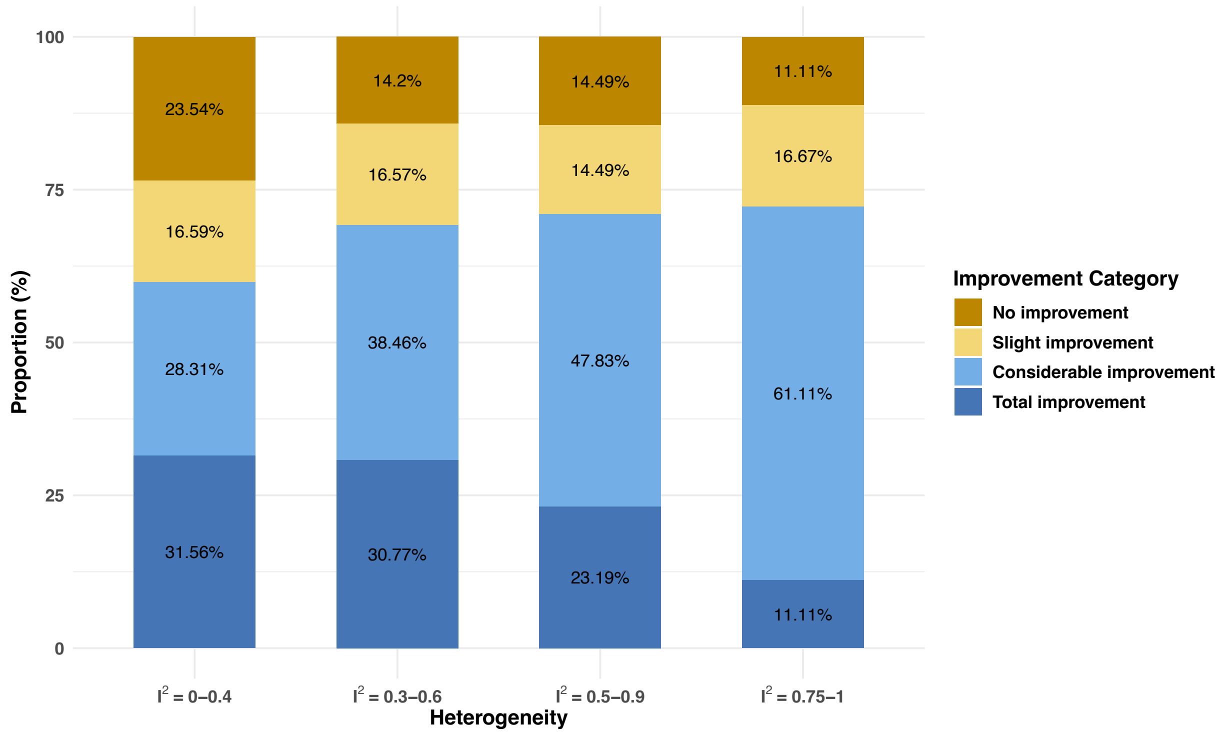

Figure S9. The improvement proportions stratified by I^2 among statistically significant meta-analyses based on scenario 2 (the REML estimator and the conventional normality-based method for deriving CIs), with RR as the effect measure.

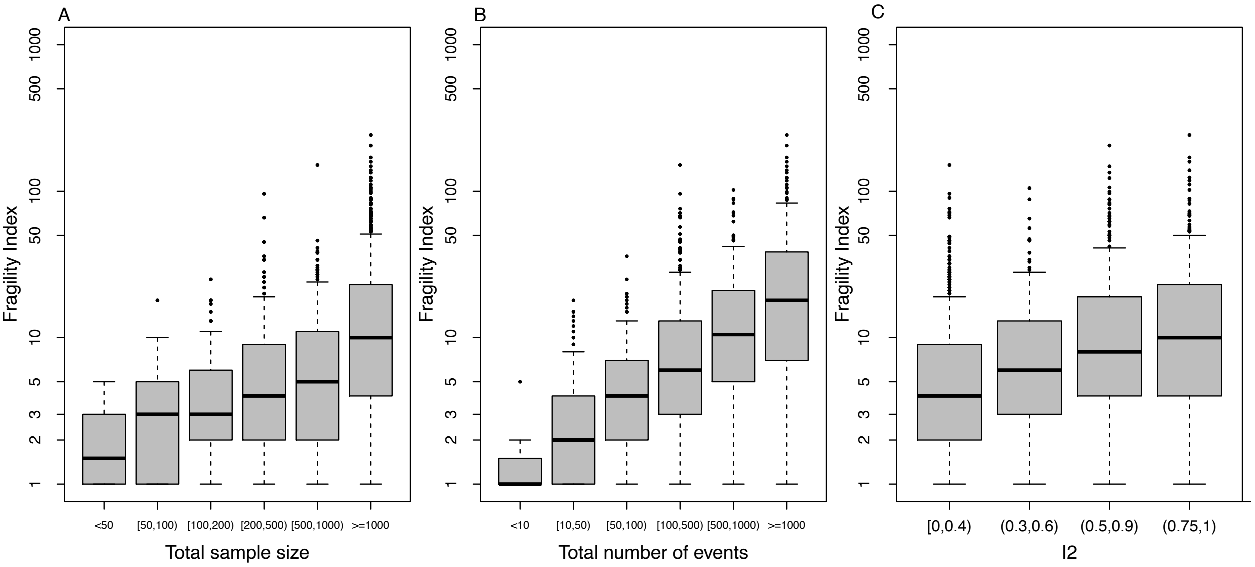

Figure S10. The FI categorized by total sample size (A), total number of events (B), and I^2 (C) for statistically significant meta-analyses based on scenario 2 (the REML estimator and the conventional normality-based method for deriving CIs), with RD as the effect measure. Total sample size and total number of events correspond to the sum of the sample sizes and the number of events in the trials included in the meta-analyses, respectively. The FI is presented on a logarithmic scale, and the analysis is limited to MAs with FI≤1000.

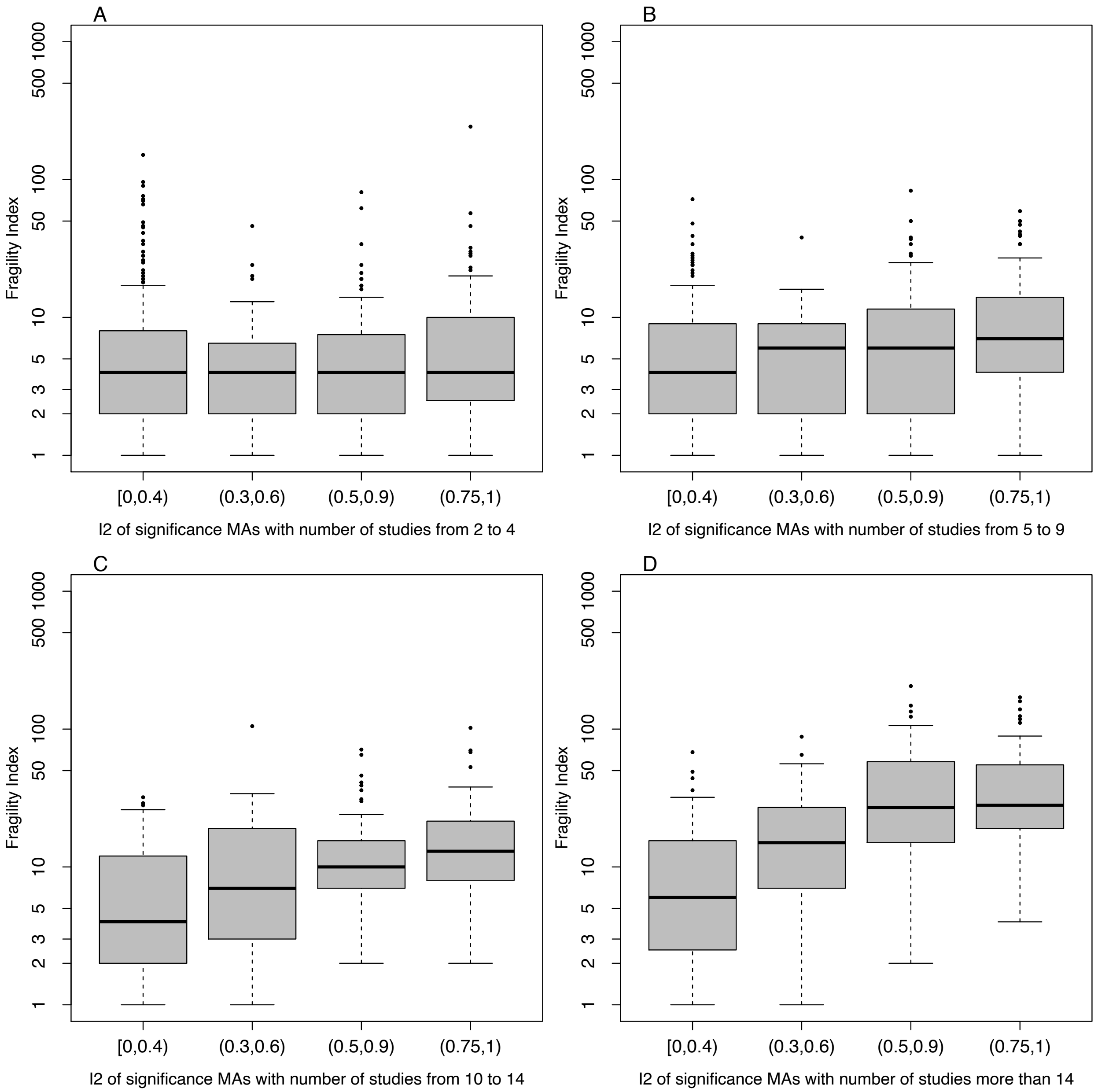

Figure S11. The FI categorized by I^2 in four subgroups based on the number of studies in scenario 2 (the REML estimator and the conventional normality-based method for deriving CIs), with RD as the effect measure.

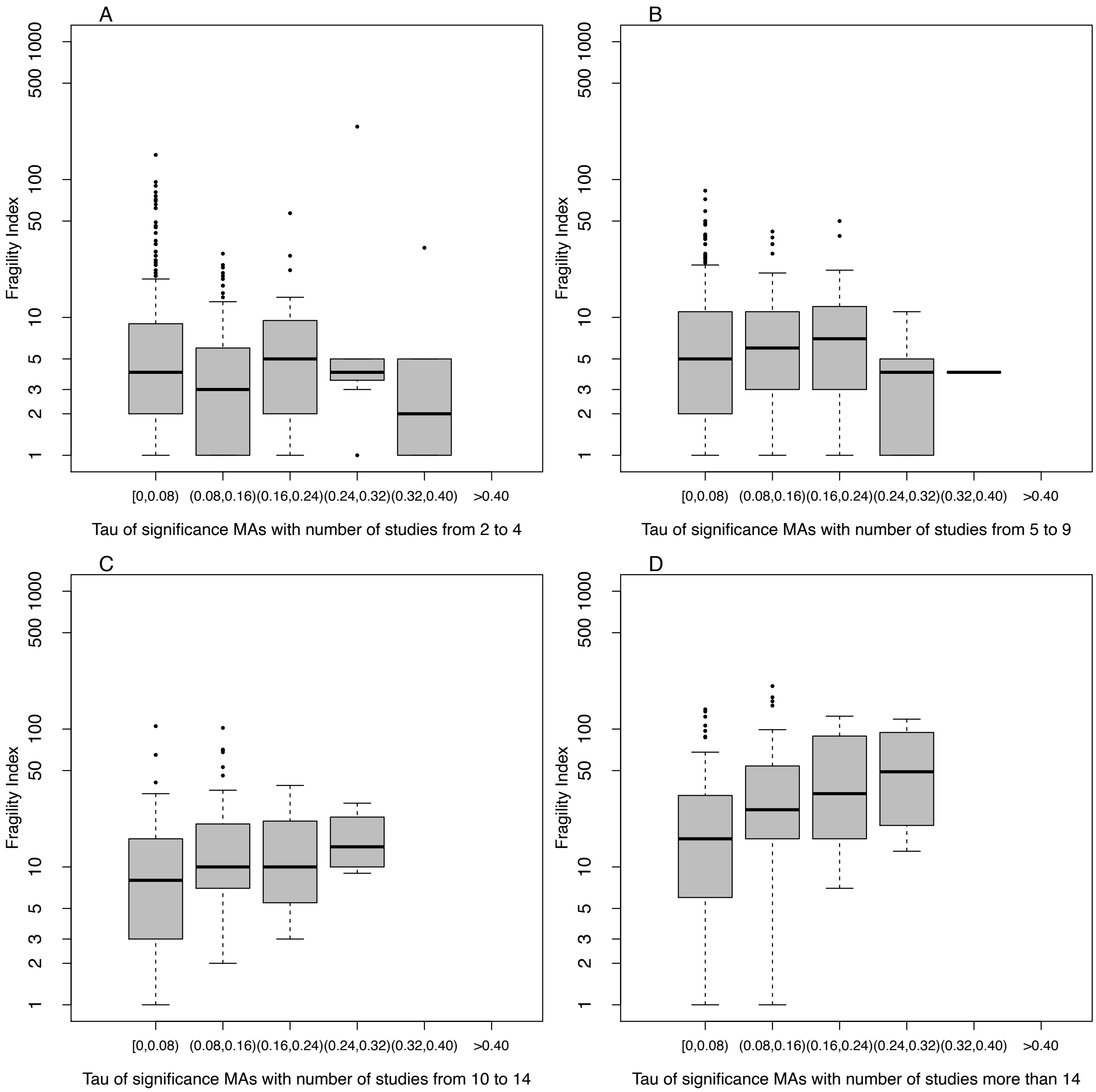

Figure S12. The FI categorized by the between-study standard deviation τ in four subgroups based on the number of studies in scenario 2 (the REML estimator and the conventional normality-based method for deriving CIs), with RD as the effect measure.

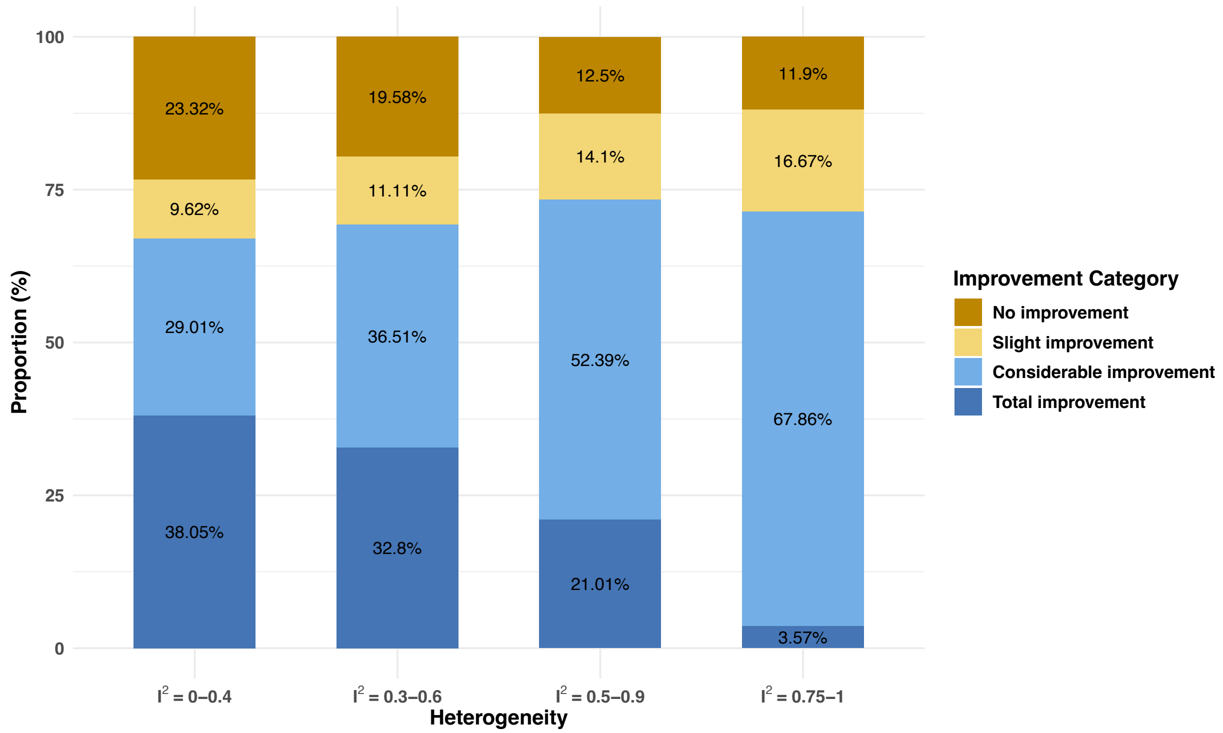

Figure S13. The improvement proportions stratified by I^2 among statistically significant meta-analyses based on scenario 2 (the REML estimator and the conventional normality-based method for deriving CIs), with RD as the effect measure.
