# Supplementary material for: Evaluating the properties of the fragility index of meta-analyses
Source: BMC Med Res Methodol. 2025 Sep 25;25:212. doi: 10.1186/s12874-025-02648-5 (PMC12465983; doi:10.1186/s12874-025-02648-5)
Supplement: Supplementary file 5 — Additional file 5: Results for meta-analyses using the fixed-effect model. [file 12874_2025_2648_MOESM5_ESM.pdf]

Additional File 5 for
“Evaluating the properties of the fragility index in meta-analyses”

Results for meta-analyses using the fixed-effect model

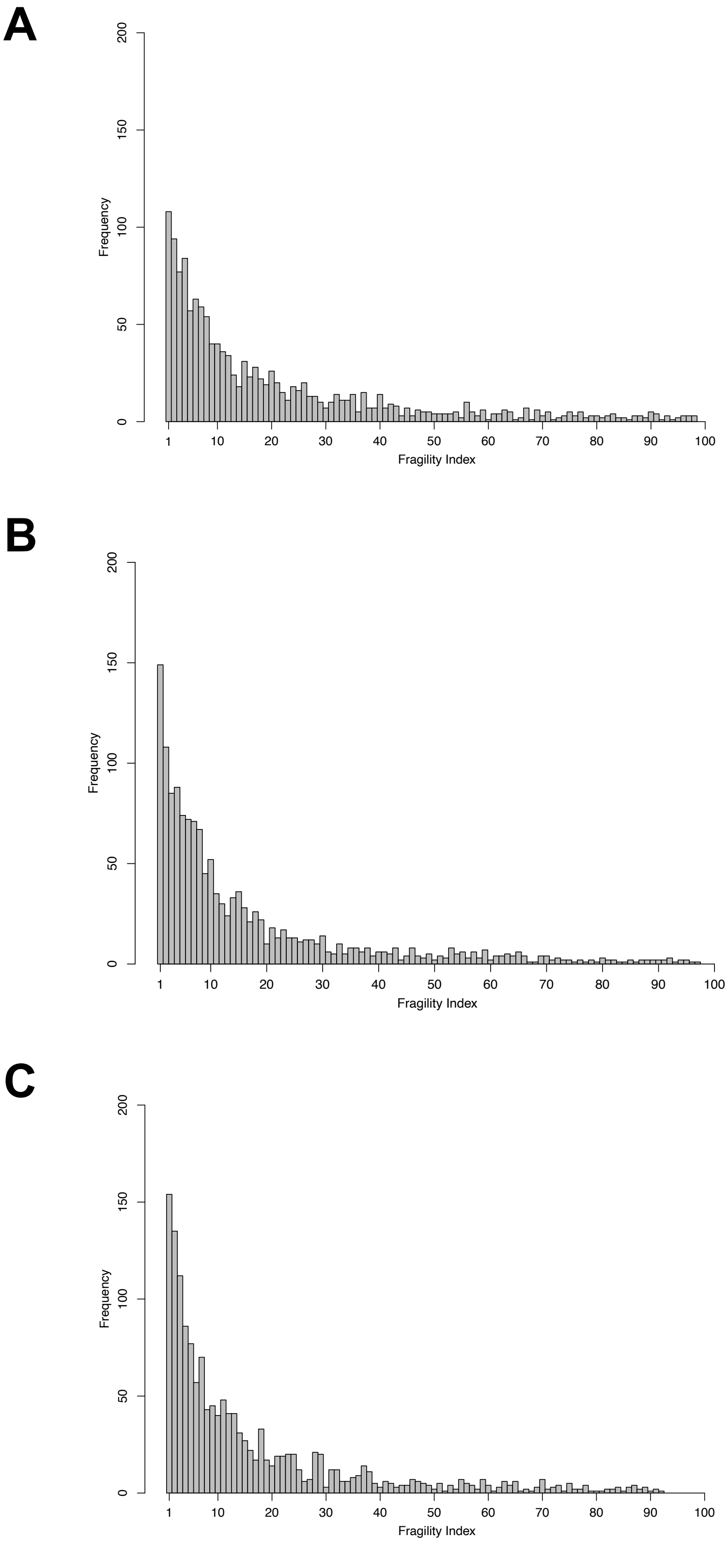

Figure S1. Histogram of the empirical distribution of FI for significant meta-analyses using the FE model, with OR (A), RR (B), and RD (C) as the effect measure.

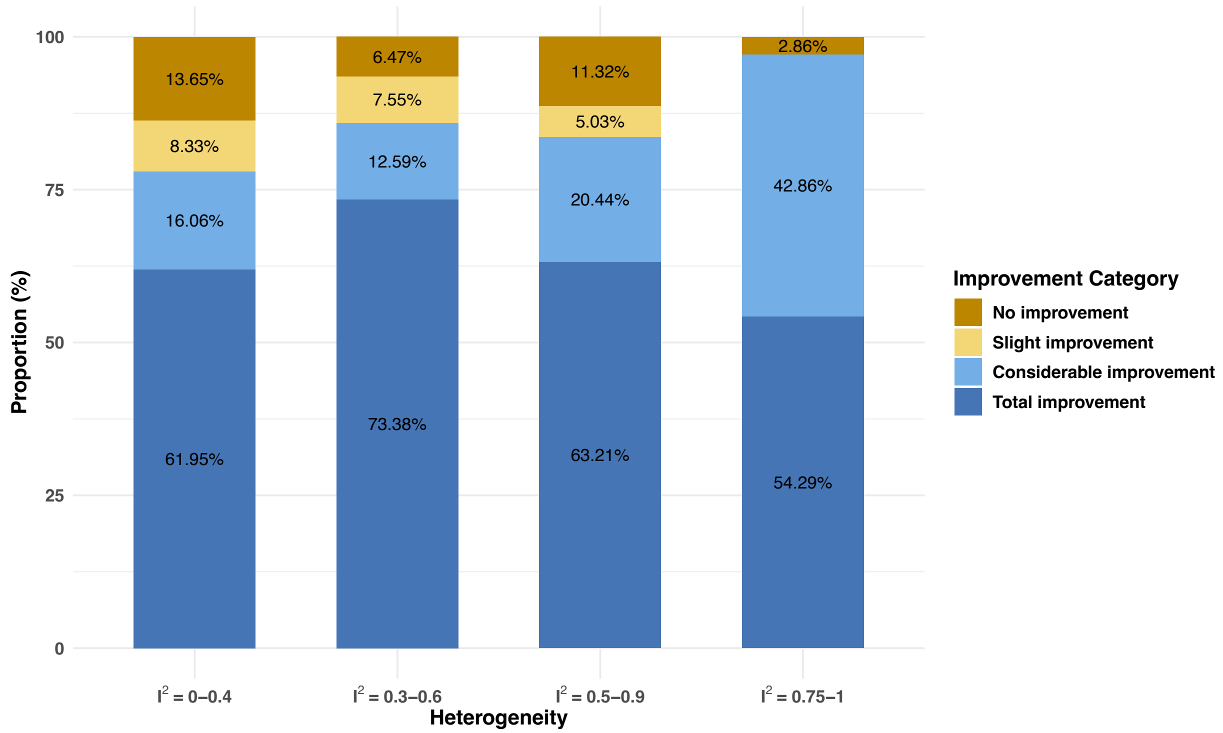

Figure S2. The improvement proportions stratified by I^2 among statistically significant meta-analyses based on the FE model, with OR as the effect measure.

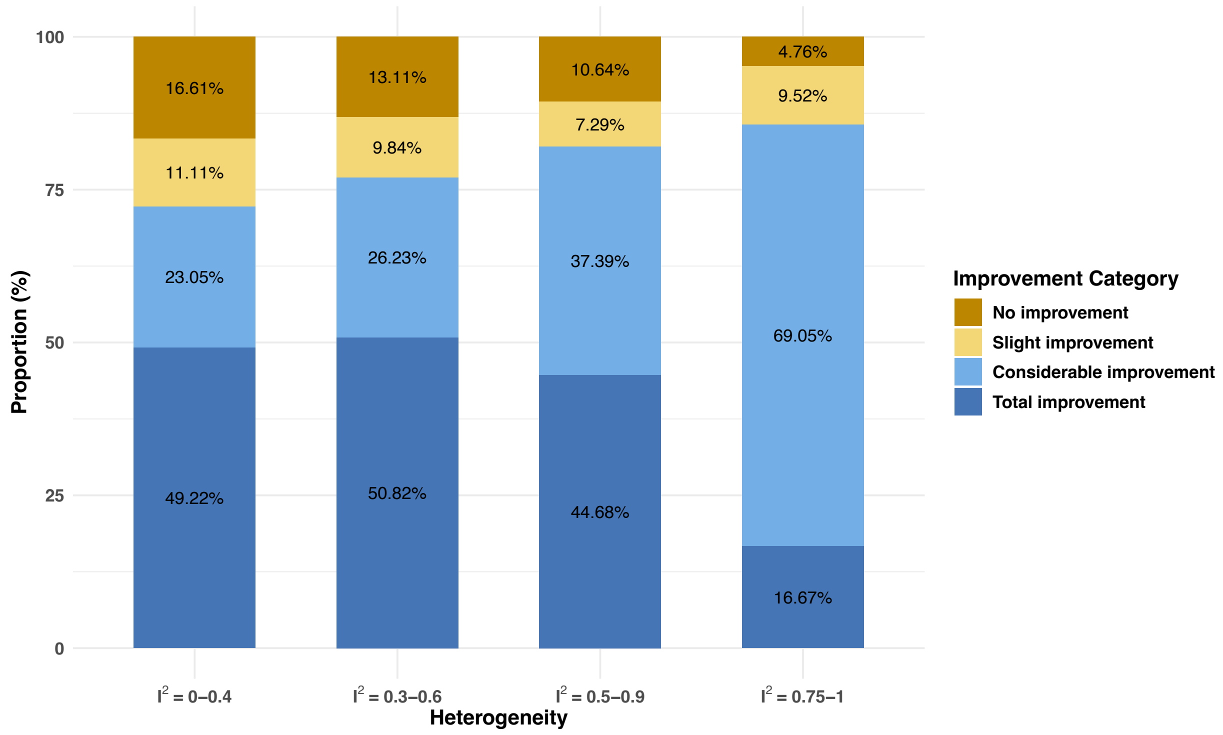

Figure S3. The improvement proportions stratified by I^2 among statistically significant meta-analyses based on the FE model, with RR as the effect measure.

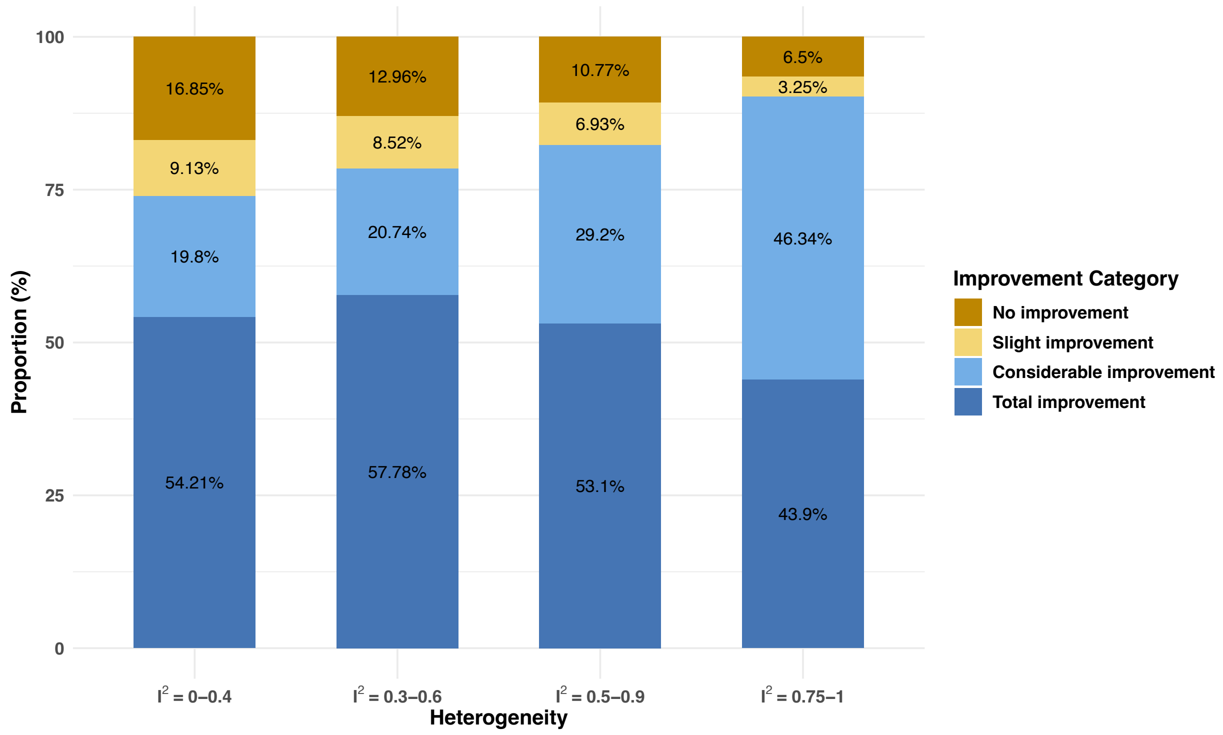

Figure S4. The improvement proportions stratified by I^2 among statistically significant meta-analyses based on the FE model, with RD as the effect measure.
